# Serjanic Acid Glycosides from *Chenopodium hybridum* L. with Good Cytotoxicity and Selectivity Profile against Several Panels of Human Cancer Cell Lines

**DOI:** 10.3390/molecules26164915

**Published:** 2021-08-13

**Authors:** Karolina Grabowska, Łukasz Pecio, Agnieszka Galanty, Paweł Żmudzki, Wiesław Oleszek, Irma Podolak

**Affiliations:** 1 Department of Pharmacognosy, Jagiellonian University Medical College, 9 Medyczna Str., 30-688 Cracow, Poland; karolina1.grabowska@uj.edu.pl (K.G.); agnieszka.galanty@uj.edu.pl (A.G.); 2 Department of Biochemistry and Crop Quality, Institute of Soil Science and Plant Cultivation–State Research Institute, ul. Czartoryskich 8, 24-100 Puławy, Poland; lpecio@iung.pulawy.pl (Ł.P.); wo@iung.pulawy.pl (W.O.); 3 Department of Medicinal Chemistry, Jagiellonian University Medical College, 9 Medyczna Str., 30-688 Cracow, Poland; zmudzki.p@gmail.com

**Keywords:** *Chenopodium hybridum*, saponins, serjanic acid, cytotoxicity

## Abstract

Two triterpene saponins, including a novel serjanic acid derivative, were isolated from *Chenopodium hybridum* L. (Amaranthaceae) aerial parts. Their structures were elucidated by a combination of spectroscopic methods (MS, 1D and 2D NMR). Both compounds were evaluated for cytotoxicity and selectivity on skin, prostate, gastrointestinal, thyroid and lung cancer cells. Their effect was dose and time-dependent with varied potency, the highest against prostate PC3 and melanoma WM793, where IC_50_ was lower than the reference drug doxorubicin. Structure–activity relationship is briefly discussed.

## 1. Introduction

The search for natural compounds that would provide an effective medicine to combat cancer is an on-going effort of many scientists. The plant kingdom offers a wide reservoir of potential drug candidates that reveal significant activity in in vitro models of cancer cell lines. Triterpene saponins are one of the most extensively studied groups of natural metabolites that were reported to exert cytotoxicity via various mechanisms including apoptotic or non-apoptotic pathways [1]. These compounds are distributed in many plant families, including Amaranthaceae, which is a source of some commercially important species like *Beta vulgaris* or *Chenopodium quinoa*. The latter is a valued crop and food product for humans and animals. Saponins identified in various plants of the Amaranthaceae family represent diverse structural types, including mono- and bidesmosides of oleanolic acid and its hydroxy-derivatives, hederagenin, sophradiol, gipsogenin, saikogenins, phytolaccagenic, akebenoic, spergulagenic and serjanic acids [2,3]. While serjanic acid is an aglycone typical of the *Phytolacca* genus (Phytolaccaceae), compounds with this genin are seldom reported in plant sources and their distribution seems to be rather scattered across families. Serjanic acid glycosides have been identified to date in *Diploclisia galucescens* (Bl.) Diels (Menispermaceae) [4], *Dendrobangia boliviana* Rusby (Cardiopteridaceae) [5] and *Phytolacca* such as *P. isocandra* L’Hérit (Phytolaccaceae) [6]. As far as the *Chenopodium* genus is concerned, this sapogenin was reported solely in two species, *Chenopodium quinoa* Willd. [3,7] and *Ch. berlandieri* spp. [8].

In the search for bioactive compounds from the *Chenopodium* genus we have investigated *Ch. hybridum* L., commonly known as maple-leaved goosefoot. The plant is an annual herb, 0.4–2 m tall, distributed across temperate regions of Asia, North America and Europe, where it was used in folk medicine as a painkiller [9]. Previous phytochemical investigations of *Ch. hybridum* were focused on flavonoids and phenolic acids [9,10,11]. Moreover, anti-oxidative, anti-inflammatory, anti-hyaluronidase and anti-proliferative properties of plant extracts were reported [10,11].

As triterpene saponins are commonly found throughout the *Chenopodium* genus this study was centered on isolation and structure elucidation of previously undescribed compounds from this group in maple-leaved goosefoot. As a result, two serjanic acid derivatives were reported in this plant species for the first time, namely 3-*O*-α-l-rhamnopyranosyl-(1→2)-α-l-arabinopyranosyl serjanic acid 28-*O*-β-d-glucopyranosyl ester and 3-*O*-α-l-arabinopyranosyl serjanic acid 28-*O*-β-d-glucopyranosyl ester [7]. The former is a novel compound, the structure of which was fully characterized based on detailed analysis of spectral data, including two-dimensional Nuclear Magnetic Resonance (2D NMR) experiments and high-resolution electrospray ionization mass spectrometry (HR-ESI-MS). Taking into account data on cytotoxic activity of triterpene saponins as well as reports on such activity observed for extracts obtained from *Ch. hybridum*, both serjanic acid glycosides were further evaluated for their cytotoxic activity and selectivity towards human cancer cell lines, grouped into four panels (skin, prostate, gastrointestinal, thyroid) as well as lung cancer.

## 2. Results and Discussion

Fruits, leaves, flowers and roots of *Ch. hybridum* L. were extracted sequentially with chloroform and methanol. Preliminary TLC comparison of methanolic extracts revealed the presence of saponins with similar chromatographic patterns in extracts from the aerial parts. Therefore, in order to isolate saponins, extract from *Ch. hybridum* aerial parts was prepared and fractionated by a combination of medium pressure liquid chromatography (MPLC) and open column chromatography (CC) on silica gel to afford two compounds, which gave a positive Liebermann–Burchard test and were isolated with purity of over 95% as confirmed by liquid chromatography-mass spectrometry (LC-MS).

**Figure 1:**
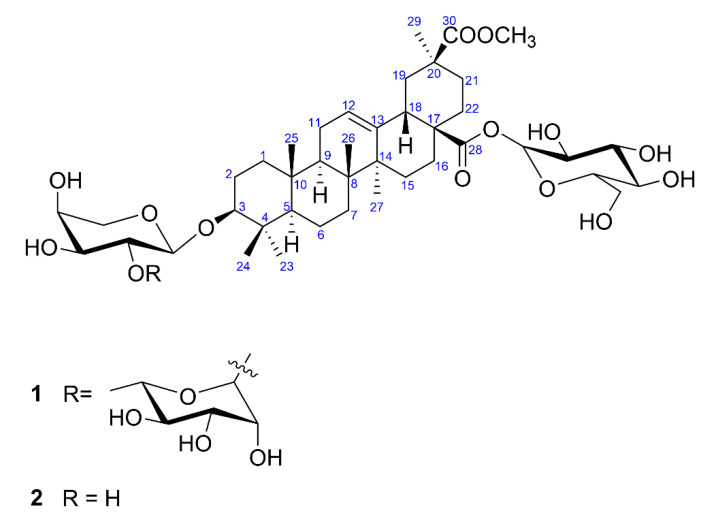
Structures of compounds **1** and **2**.

Compound **1** (Figure 1) was obtained as a white powder. Its molecular formula was determined from the HR-ESI-MS spectrum, which gave a peak at *m*/*z* 963.4929 [M + Na]^+^ (calculated for C_48_H_76_O_18_Na). The negative ion mode ESI-MS showed peaks at *m*/*z* 985.49 and *m*/*z* 939.49 which were assigned to an adduct [M + HCOO]^−^ and ion [M-H]^−^, respectively. MS^2^ fragmentation of these ions indicated the loss of hexose and deoxyhexose units at *m*/*z* 631.45 [M-H-162-146]^−^. Moreover, MS^2^ in positive ion mode showed a peak at *m*/*z* 483.30 corresponding to an additional loss of pentose and dehydration of the sapogenin [M + H-162-146-132-18]^+^. The obtained data agreed with the results of acidic hydrolysis of compound **1**, which afforded glucose, rhamnose and arabinose, identified by TLC chromatography with reference sugar samples.

**Figure 2:**
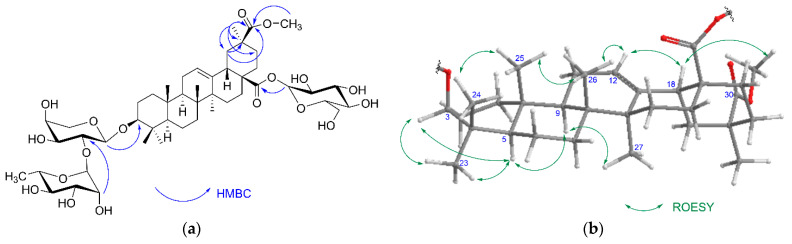
Key HMBC (**a**) and ROESY (**b**) correlations of compound **1**.

The ^1^H NMR spectrum of **1** (Table 1) showed signals for six methyl groups linked to quaternary carbon atoms at δ 0.84 (s, 3H, Me-25), 0.89 (s, 3H, Me-24), 1.04 (s, 3H, Me-26), 1.17 (s, 3H, Me-29), 1.18 (s, 3H, Me-23) and 1.26 (s, 3H, Me-27), a triplet of the olefinic proton (δ 5.59, H-12, *J* = 3.7 Hz), an oxymethine proton at δ 3.29, dd, *J* = 11.8 and 4.4 Hz, H-3, and doublet of doublets of H-18 (δ 3.14, *J* = 14.0 and 4.7 Hz) which indicated an oleanane skeleton of the sapogenin. A methoxy group at δ 3.51 (s) was also seen. The ^13^C NMR (Table 1) and Heteronuclear Single Quantum Coherence (HSQC) spectra allowed us to identify signals that confirmed an oleane-12-ene type for the aglycone, namely six methyl groups at δ 28.7 (C-23), 17.3 (C-24), 16.0 (C-25), 17.8 (C-26), 26.3 (C-27), 28.8 (C-29), a hydroxyl at C-3 (δ 89.2) and olefinic carbon signals of the double bond at δ 123.6 (C-12) and 144.3 (C-13). Two carbonyl carbons at δ 176.6 and 177.4 were assigned to C-28 and C-30, respectively, based on Heteronuclear Multiple Bond Correlation (HMBC) correlations (Figure 2) between H-18 and C-28, and H-19, H-21, Me-29, and the proton of a methoxy group (δ 3.51) and C-30. The above data were in good agreement with serjanic acid [4,5,6,7].

The linkage position of the sugar chains to the aglycone and interglycosidic linkages were established from 2D NMR spectra. The downfield shift values of C-3 (δ 89.2) and C-28 (δ 176.6) suggested that compound **1** is a 3,28-bidesmoside. The ^1^H NMR spectrum showed three doublets of anomeric protons at δ 4.75 (*J* = 6.9 Hz), 6.08 (*J* = 7.9 Hz) and 6.46 (*J* = 1.6 Hz) that corresponded to the carbon signals at δ 107.7, 95.4 and 101.9 as revealed by the HSQC experiment. Sugars were identified based on the results from acid hydrolysis of compound **1** on a TLC plate together with mass spectra fragmentation, coupling constants values and detailed analysis of ^1^H-^1^H 2D COSY (Correlation Spectroscopy), TOCSY (Total Correlation Spectroscopy), ^1^H-^13^C HMBC, H2BC (Heteronuclear two-bond Correlation) and F_2_-coupled HSQC [12] as α-arabinopyranose, β-glucopyranose and α-rhamnopyranose, respectively. The attachment of the glucose moiety at C-28 was deduced from the upfield shift of the anomeric carbon (δ 95.4) and HMBC correlation between anomeric H-1 (δ 6.08) and the carbon signal at δ 176.6 (C-28). The second sugar chain was assigned at C-3 based on HMBC correlation between the anomeric proton of arabinose at δ 4.75 and carbon signal at δ 89.2. The 3β-configuration was confirmed by the T-ROESY (Transverse Rotating frame Overhauser Enhancement Spectroscopy) experiment in which cross peaks between H-3α, H-5α and proton signal of Me-23 were seen (Figure 2). The linkage of the terminal rhamnose unit was established from the HMBC correlation between its anomeric proton at 6.46 and C-2 of α-arabinose (δ 73.1). Consequently, the structure of saponin **1** was concluded to be a new compound, determined as 3-*O*-α-l-rhamnopyranosyl-(1→2)-α-l-arabinopyranosyl serjanic acid 28-*O*-β-d-glucopyranosyl ester.

The structure of compound **2** (Figure 1) was elucidated by careful analysis of LC-MS/MS fragmentation pattern and 1D and 2D NMR spectra. The ESI-MS spectrum in negative ion mode gave an adduct at *m*/*z* 839 [M + HCOO]^−^. MS^2^ fragmentation of this ion indicated the loss of hexose (*m*/*z* = 631.45 [M-162-H]^−^). Moreover, in positive ion mode, [M-162+H]^+^, [M-162–132+H]^+^, [M-162–132–18+H]^+^ ions indicated the loss of a hexose, a pentose and dehydration, respectively. Likewise, the key 2D NMR correlations indicated that only two monosaccharide units were attached to serjanic acid, namely glucose at C-28 and arabinose at C-3. Comparison of the spectroscopic evidence with literature data confirmed that compound **2** is identical with 3-*O*-α-l-arabinopyranosyl serjanic acid 28-*O*-β-d-glucopyranosyl ester, isolated previously from *Ch. quinoa* Willd. [7]. This saponin was also reported in the grains of *Ch. berlandieri* spp. based on the results obtained from HPLC coupled with an evaporative light scattering detector (ELSD) where peaks were identified according to their similarity with *Ch. quinoa* compounds [8]. Nevertheless, it is noteworthy that both serjanic acid bidesmosides are not common within the *Chenopodium* genus, and *Ch. hybridum* as their novel source represents plant species typical of the European flora as opposed to *Ch. quinoa* and *Ch. berlandieri* which are distributed mainly in Central and South America. This may be an important factor that could contribute to observations on inter-species relationships within the *Chenopodium* genus derived from genetic studies. A phylogeny-based classification of *Chenopodium sensu lato* [13] has revealed that while *Ch. quinoa* and *Ch. berlandieri* belong to the same clade (*Chenopodium*), *Ch. hybridum* was included into *Ch. murale* clade, which was given a new name–*Chenopodiastrum*. Nevertheless, both *Chenopodium* and *Chenopodiastrum* are classified to the Atripliceae tribe. 

**Figure 3:**
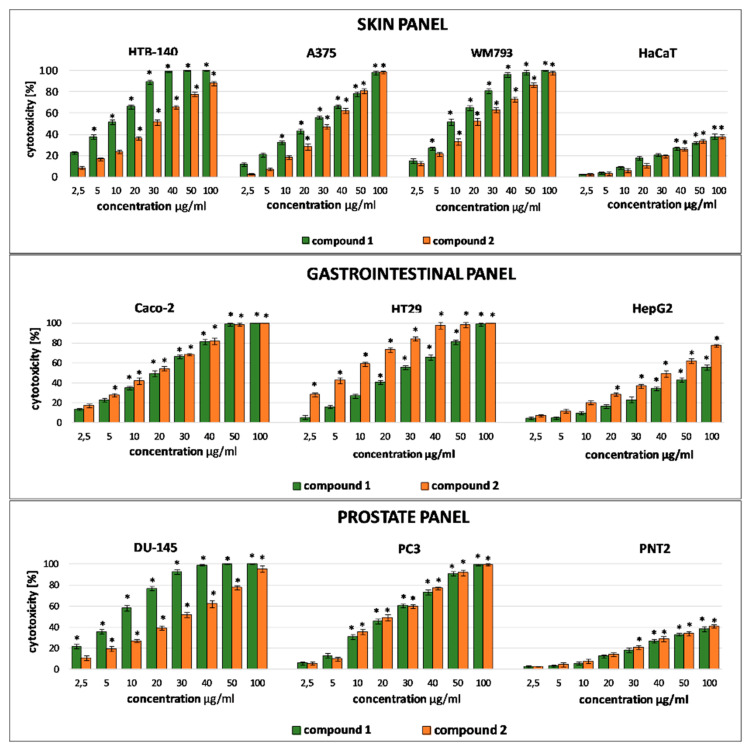
Cytotoxic activity of compounds **1** and **2** towards human cell lines, grouped into panels-skin panel: melanoma (HTB140, WM793, A375), keratinocytes HaCaT cells; gastrointestinal panel: colorectal adenocarcinomas (Caco2, HT29); hepatocellular carcinoma (HepG2); prostate panel: prostate carcinoma (DU145,PC3), normal prostate epithelial (PNT2). Results from the LDH viability assay after 24 h incubation with tested substances. Results are presented as the mean ± SD. The values significantly different from the control (untreated group) are indicated by * for *p* ˂ 0.05 (Mann–Whitney U test).

Based on literature reports confirming cytotoxic activity of crude methanolic extracts from *Ch. hybridum* [9,10] cytotoxicity of isolated compounds **1** and **2** was evaluated by the LDH assay on several human cancer and normal cell lines (Figure 3). Data expressed as IC_50_ is summarized in Table 2. Bearing in mind that phenotypic heterogeneity of tumor cells is critical to their resistance to therapy and treatment failures, cytotoxicity experiment was designed in such a way to mimic the complex nature of a tumor. Therefore, the assay included cancer cells of different origin (skin, prostate, gastrointestinal, thyroid, lung), malignancy and metastatic potential (cells from primary tumor, cells derived from metastatic sites) and, in order to verify the selectivity of the tested substances, the corresponding normal cells.

Both serjanic acid glycosides were found to act selectively with no effect observed on normal PNT2, Nthy-ori 3–1 and HaCaT cells. Their cytotoxicity towards cancer cells was varied and depended on a given tumor type. It is interesting to note that both saponins were more potent than the reference compound (doxorubicin) against primary melanoma WM793 and prostate carcinoma derived from metastatic site PC3. In all cases cytotoxic activity of **1** and **2** was dose and time-dependent. Literature data indicate that bidesmosidic saponins of plant origin, oleanane derivatives especially, are generally less potent than monodesmosides with a free carboxylic group at C-17 (C-28) [14]. On the other hand, several studies revealed that the activity of oleanane-type saponins is affected not only by the functional groups in sapogenin but also by the number and type of monosaccharide units in the sugar part [1]. Numerous reports confirmed that a rhamnose unit in the sugar chain attached at C-3 is a structural feature that enhances cytotoxicity of triterpene saponins [15]. Moreover, a sugar chain that incorporates a monosaccharide unit composed of α-l-rhamnopyranosyl-(1→2)-α-l-arabinopyranosyl located at C-3 is also considered to be a key factor for cytotoxic activity of oleanane type saponins [14,16]. Such a unique disaccharide sequence located at C-3 of serjanic acid may be responsible for the observed activity of compound **1** towards cells grouped in the skin panel (HTB-140, A375, WM793) where the activity of compound **1** was higher as compared to **2**, which is devoid of the terminal rhamnose unit. However, this structure-activity correlation is not evident in other types of cancer cells. Compound **2** was found to be more effective towards gastrointestinal cells (Caco-2, HepG2, HT29) than compound **1**. Cytotoxicity data available in the literature have shown that compound **2**, as well as other serjanic acid bidesmosidic saponins isolated from *Ch. quinoa* were inactive towards HeLa cells [7].

A lack of influence on the viability of normal prostate cells and skin keratinocytes observed in our study is also noteworthy. In another experiment, other serjanic acid bidesmosidic saponins with glucuronic acid and rhamnose in the sugar chain attached at C-3 were found to be cytotoxic towards human skin fibroblasts with IC_50_ 5.6–6.4 μM [5].

Taken together, data from our experiment show that the structure of saponin influences the cytotoxicity and selectivity, but the overall effect depends mainly on the susceptibility of cells which represent a given tumor type.

## 3. Materials and Methods

### 3.1. General Experimental Procedures

NMR experiments 1D (^1^H NMR–500 MHz, ^13^C NMR–125 MHz) and 2D (HSQC, F_2_-coupled HSQC, H2BC, HMBC, COSY, TOCSY, T-ROESY) for compound **1** were performed on a BrukerAvance III HD Ascend-500 spectrometer (Bruker BioSpin, Rheinstetten, Germany), equipped with 5 mm ^1^H{^109^Ag-^31^P} broad-band inverse (BBI) probe; spectra were recorded in the mixture of pyridine-d_5_/D_2_O (250/10) with 0.2% TFA. Spectra for compound **2** were recorded in methanol-d_4_ on JNM-ECZR500 RS1 500 MHz (JEOL), using standard pulse sequence at 500 MHz.

Chemical shifts (δ) are given in ppm. Coupling constants are reported in Hz. High resolution mass spectra were obtained on a high-resolution quadrupole time-of-flight mass spectrometer (HR/Q-TOF/MS, Impact II HD, Bruker Daltonik GmbH, Bremen, Germany) operated in the negative and positive electrospray ionization mode with mass scan range set from *m*/*z* 375 to *m*/*z* 2200, using capillary voltage of 3.0 and 4.5 kV, respectively; nitrogen (N_2_) was used as nebulizer and drying gas at 2.0 bar and 6.0 L min^−1^, respectively; dry temperature was set to 200 °C. The MS/MS spectra were acquired using variable collision energy in the range from 10 to 100 eV. Internal calibration of the instrument was accomplished using 10 mM sodium formate solution introduced to the ion source via a 20 μL loop at the beginning of each analysis. Data were collected and processed by the Data Analysis 4.4 software (Bruker Daltonik GmbH, Bremen, Germany).

LC-MS/MS analysis was performed on UPLC/MS Waters ACQUITY TQD (Waters Corporation, Milford, MA, USA) apparatus with gradient elution (95% to 0% of eluent A; eluent A: water/formic acid (0.1%, *v*/*v*); eluent B: acetonitrile/formic acid (0.1%, *v*/*v*)). Chromatographic separations were carried out using the Acquity UPLC BEH (bridged ethyl hybrid) C18 column; 2.1 × 100 mm, and 1.7 µm particle size, equipped with Acquity UPLC BEH C18 VanGuard pre-column; 2.1 × 5 mm, and 1.7 µm particle size. Chromatograms were recorded using Waters eλ PDA detector. Spectra were analyzed in 200–700 nm range with 1.2 nm resolution and sampling rate 20 points/s.

MS detection settings of Waters TQD mass spectrometer were as follows: source temperature 150 °C, desolvation temperature 350 °C, desolvation gas flow rate 600 L h^−1^, cone gas flow 100 L h^−1^, capillary potential 3.00 kV and cone potential 30 V. Nitrogen was used for both nebulizing and drying gas. The data were obtained in a scan mode ranging from 50 to 1000 *m*/*z* in 0.5 s time intervals. 

Medium Pressure Liquid Chromatography (MPLC) was performed on a Sepacore apparatus (BÜCHI Labortechnik AG, Flawil, Switzerland) with a C-615 Pump Manager and C-660 Fraction Collector, column 15.0 × 4.0 cm. 

Open column chromatography and MPLC was carried out on silica gel 230–400 mesh (Sigma-Aldrich, Darmstadt, Germany), thin-layer chromatography (TLC) was performed on pre-coated silica gel 60 plates (0.25 mm Merck), preparative thin-layer chromatography (pTLC) was carried out on commercial glass Silica Gel G (500 μ) ANALTECH. Spraying reagents for saponins: 25% solution of H_2_SO_4_ in methanol and heating at 120 °C on a TLC plate heater III (CAMAG, Muttenz, Switzerland) for 3 min.; for sugars: aniline phthalate reagent and heating at 100°C for 5 min on a TLC plate heater III (CAMAG, Muttenz, Switzerland). Cytotoxicity assay was performed using a Microplate Reader (BioTek Instruments Inc., Winooski, VT, USA) equipped with Gen 5 software. All reagents used were of analytical grade. Reference standards of authentic sugars were from (Sigma-Aldrich, Germany).

### 3.2. Plant Material

*Chenopodium hybridum* L. was collected from natural stands in Cracow, Poland (50.06262, 19.92971). Botanical identification of the species was carried out by a botanist from the Department of Pharmacognosy UJCM. A voucher specimen (No. KFg/ChHbr-2019) is deposited at the Department of Pharmacognosy, Pharmaceutical Faculty, Medical College, Jagiellonian University, Cracow, Poland.

Plant material was air-dried under steady, controlled conditions (at room temperature, in the dark) and divided into aerial part and roots. Aerial parts of the herb were separated into leaves, flowers, fruits and stems. Plant material was stored in airtight containers.

### 3.3. Extraction and Isolation

Dried plant material (aerial parts) (leaves 10 g, fruits 10 g, flowers 10 g, stems 10 g, roots 10 g) of *Ch. hybridum* were ground to a fine powder using a laboratory mill (BOSCHMKM6003, BSHGmbH, Munich, Germany) and extracted successively with chloroform (plant material/solvent ratio-DSR 1:10 *w*/*v*, 2× for 2 h and methanol, 3× for 3 h, DSR 1:10) on a boiling water bath under reflux. Evaporation of methanol under reduced pressure on a rotary evaporator gave dark brown crude extracts from leaves (65 mg), fruits (125 mg), flowers (45 mg) and stems (38 mg), which were subjected to TLC (silica gel, CHCl_3_-MeOH-H_2_O (23:12:2 *v*/*v*), 25% H_2_SO_4_ in MeOH + heating). Preliminary TLC analysis revealed a similar chromatographic pattern in all morphotic aerial parts. In order to isolate saponins, extracts from a larger sample of aerial parts of *Ch. hybridum* (200 g) was prepared in an analogous manner as described above. The MeOH extract was concentrated in vacuo to yield 14.8 g residue. Extract portions (3.0 g) were chromatographed by MPLC on silica gel (MPLC column 150 × 40 mm, flow rate 10 mL/min) using isocratic solvent system CHCl_3_/MeOH/H_2_O (23:12:2) to give 7 fractions (I-VII) which were further purified by repeated column chromatography (CC column: 80 × 12 mm) under the same conditions and preparative thin layer chromatography in CHCl_3_/MeOH/H_2_O (27:10:2) to yield compound **1** (17 mg from fr. V) and compound **2** (23 mg from fr. III).

### 3.4. Acid Hydrolysis

Acid hydrolysis of isolated **1** and **2** was performed on TLC plates using HCl *in statu nascendi* for 30 min at 60°C. The plates were developed twice in CHCl_3_–MeOH–H_2_O (23:12:2 *v*/*v*) together with sugar standards (d-glucose R_f_ = 0.32; d-galactose R_f_ = 0.28; l-arabinose R_f_ = 0.37; l-rhamnose R_f_ = 0.51; d-xylose R_f_ = 0.42; glucuronic acid lactone R_f_ = 0.6). Chromatograms were visualized by spraying the TLC plates with aniline phthalate and heating (120 °C, 20 min) [17].

### 3.5. Cell Culture

Human cancer and the corresponding normal cell lines, used in the study, were grouped as follows: skin panel (melanoma HTB140, derived from metastatic site: lymph node, ATCC Hs 294T; malignant melanoma A375, ATCC CRL-1619; stage I primary melanoma WM793, RRID:CVCL 8787; skin keratinocytes HaCaT), prostate panel (prostate carcinoma Du145, derived from metastatic site: brain, ATCC HTB-81; grade IV prostate carcinoma, PC3, derived from metastatic site: bone, ATCC CRL-1435; prostate epithelial cells PNT2, ECACC 95012613), gastrointestinal panel (colorectal adenocarcinomas Caco2, ATCC HTB-37, and HT29, ATCC HTB-38; hepatocellular carcinoma HepG2, ATCC HB-8065), thyroid panel (follicular thyroid carcinoma FTC133, ECACC 94060901; undifferentiated thyroid carcinoma 8505C, ECACC 94090184; Nthy-ori 3–1, human thyroid follicular epithelial cells, ECACC 90011609), lung carcinoma A549 cells (ATCC CRM-CCL-185). Cells were grown at standard conditions (37 °C, 5% CO_2_, relative humidity) and culture media (DMEM/F12 for PNT2, WM 793, HT29, PC3, FTC133, 8505C, A549; DMEM Low Glucose for DU145; DMEM High Glucose for HTB140, A375, HaCaT; MEM with NEAA for Caco2), supplemented with 10% fetal bovine serum (FBS) and 1% antibiotics solution (10 000 U penicillin and 10 mg streptomycin/mL). All culture media and supplements were from Sigma-Aldrich.

### 3.6. Cytotoxic Activity Assay

Cell viability was measured with the lactate dehydrogenase (LDH) assay (Clontech, Mountain View, CA, USA), as described previously [18]. Before the experiment, cells were seeded onto 96-well plates for 24 h (1.5 × 104 cells/well). The culture medium was replaced with a fresh medium containing different concentrations of the tested extracts (10–100 μg/mL, in DMSO) and incubated for 24 and 48 h. The absorbance was measured at 490 nm using a Biotek Synergy microplate reader. Cell viability was expressed as percent of the dead cells and IC_50_ values were also calculated. Each experiment was done in triplicate.

### 3.7. Data Analysis

Data were analyzed using Statistica v. 13.3 (Stat Soft, TIBCO, Palo Alto, CA, USA). Each variable was expressed as mean (± SD). The statistical significance in cytotoxic assay was determined using Mann–Whitney U test. The value *p* < 0.05 was considered to indicate significant differences. NMR Spectra were analyzed using ACD/Labs ^1^D NMR Processor 12.0 (Academic Edition) and JEOL DELTA v 5.3.1 (Jeol Resonance Inc., JEOL, Tokyo, Japan). In MS analysis MassLynx V 4.1 (Waters, Milford, MA, USA) and Compass DataAnalysis (Bruker, Billerica, MA, USA) software were used. IC_50_ values in cytotoxic assay were calculated using Origin Pro 2020b (OriginLab Corporation, Northampton, MA, USA). 

### 3.8. 3-O-α-l-Rhamnopyranosyl-(1→2)-α-l-Arabinopyranosyl Serjanic Acid 28-O-β-d-Glucopyranosyl Ester (Compound ***1***)

White powder; ^1^H and ^13^C NMR: see Table 1. MS: HRESIMS *m*/*z* 963.4929 [M+Na]^+^(calcd. for C_48_H_76_O_18_Na).

ESI MS (negative ion mode) *m*/*z* 939 [M-H]^−^; *m*/*z* 985.49 [M + HCOO]^−^, fragmentation in MS/MS: *m*/*z* 631.45 [M-H-162–146]^−^; (positive ion mode) *m*/*z* 958 [M + NH_4_]^+^, fragmentation in MS/MS, *m*/*z* 483.30 [M + H-162–146–132–18]^+^.

Spectra are provided in the Supplementary Material.

### 3.9. 3-O-α-l-Arabinopyranosyl Serjanic Acid 28-O-β-d-Glucopyranosyl Ester (Compound ***2***)

White powder; ^1^H and ^13^C NMR: see Table S1 (Supplementary Materials). MS: HRESIMS *m*/*z* [M+Na]^+^ (calcd. for C_42_H_66_O_14_Na, 817.4350). 

ESI MS (negative ion mode) *m*/*z* 839.45 [M + HCOO]^−^, fragmentation in MS/MS: *m*/*z* 631.45 [M-H-162]^−^; (positive ion mode) *m*/*z* 812 [M + NH_4_]^+^, fragmentation in MS/MS: *m*/*z* 650.23 [M + NH_4_ -162]^+^, *m*/*z* 633.01 [M+ H -162]^+^, *m*/*z* 501 [M + H- 162–132]^+^, *m*/*z* 483.30 [M + H-162–132–18]^+^,

Spectra are provided in the Supplementary Material.

## 4. Conclusions

*Chenopodium hybridum* is a novel source of serjanic acid glycosides, including a new compound, the structure of which was elucidated as 3-*O*-α-l-rhamnopyranosyl-(1→2)-α-L-arabinopyranosyl serjanic acid 28-*O*-β-d-glucopyranosyl ester. This sapogenin has been described for the first time in a representative of *Chenopodium* genus typical of European flora. Both isolated bidesmosides showed interesting cytotoxic activity, selectively and dose- and time-dependently inhibiting the growth of several human cancer cell lines and were found more potent than the reference compound (doxorubicin) against primary melanoma WM793 and prostate carcinoma derived from metastatic site PC3. 

## Figures and Tables

**Table 1:** ^1^H (500 MHz) and ^13^C (125 MHz) NMR spectral data (δ ppm) for saponin **1** (pyridine-d_5_/D_2_O 250:10 with 0.2% trifluoroacetic acid).

No.	δ_C_	δ_H_ (*J* in Hz) *
1	39.3	0.92 t (12.4), 1.50 d (13.4)
2	27.0	1.83 q (13.00), 2.13 d (13.7)
3	89.2	3.29 dd (11.8, 4.4)
4	39.9	-
5	56.4	0.74 d (11.5)
6	19.0	1.26, 1.41
7	33.7	1.43, 1.66 d (13.0)
8	40.3	-
9	48.5	1.69 dd (10.9, 6.8)
10	37.4	-
11	24.2	1.89
12	123.6	5.59 t (3.7)
13	144.3	-
14	42.6	-
15	29.2	1.64, 2.04
16	23.9	2.21
17	47.2	-
18	43.9	3.14 dd (14.0, 4.7)
19	42.9	1.80 t (13.7), 2.21 dd (12.9, 3.9)
20	44.3	-
21	31.0	1.40 dd (13.5, 4.0), 2.08
22	34.0	1.94
23	28.7	1.18 s
24	17.3	0.89 s
25	16.0	0.84 s
26	17.8	1.04 s
27	26.3	1.26 s
28	176.6	-
29	28.8	1.17 s
30	177.4	-
-OCH_3_	52.1	3.51 s
3-*O*-α-L-Ara		
1	107.7 (^1^*J*_CH_ 158)	4.75 d (7.9)
2	73.1	4.40 dd (8.7, 6.9)
3	74.8	4.16 dd (8.8, 3.5)
4	69.7	4.32
5	66.9	3.81 dd (12.1, 1.6)
α-L-Rha		4.29 dd (12.1, 3.5)
1	101.9 (^1^*J*_CH_ 172)	6.46 d (1.6)
2	72.4	4.77 dd (3.5, 1.6)
3	72.8	4.53 dd (9.4, 3.5)
4	74.1	4.30 t (9.4)
5	70.2	4.50 dq (9.4, 6.1)
6	19.0	1.73 d (6.2)
28-*O*-β-D-Glc		
1	95.4 (^1^*J*_CH_ 166)	6.08 d (7.9)
2	76.2	4.39 t (8.2)
3	79.7	4.28
4	71.5	4.28
5	79.2	3.92 ddd (9.4, 4.1, 2.6)
6	62.1	4.22 dd (12.0, 4.2)4.26 dd (12.0, 2.8)

* Overlapping signals are reported without designated multiplicity.

**Table 2:** Cytotoxicity of serjanic acid glycosides **1** and **2** isolated from *Chenopodium hybridum* L.

Cell Line	IC_50_ [µg/mL]				
	Compound 1		Compound 2		Doxorubicin
	24 h	48 h	24 h	48 h	24 h
HTB-140	11.66	7.95	39.24	25.09	5.71
A375	32.42	20.34	39.37	29.28	0.59
WM793	17.22	9.92	32.24	16.76	>40
HaCaT	>100	>100	>100	>100	4.68
DU-145	16.63	7.25	33.13	23.53	3.18
PC3	34.28	19.46	23.81	18.27	>50
PNT2	>100	>100	>100	>100	1.38
FTC133	>100	76.30	>100	>100	4.02
8505C	>100	>100	>100	>100	>40
Nthy-ori 3–1	>100	>100	>100	>100	
Caco-2	28.72	15.28	20.72	12.57	3.44
HT29	57.86	22.77	10.66	6.48	1.53
HepG2	>100	76.62	54.20	38.96	1.03
A549	61.32	34.74	41.70	28.27	1.73

Abbreviations: melanoma: HTB140, WM793, A375; keratinocytes HaCaT cells; colorectal adenocarcinomas: Caco2, HT29; hepatocellular carcinoma: HepG2; prostate carcinoma: DU145, PC3; normal prostate epithelial: PNT2; FTC133, 8585C, thyroid carcinoma: FCT133, 8505C; human thyroid follicular epithelial cells: Nthy-ori3–1; lung carcinoma: A549.

## Supplementary Materials

The following are available online. Figure S1.^1^H NMR (500 MHz, pyridine-d_5_/D_2_O (250/10) with 0.2% TFA) spectrum of compound **1**, Figure S2: ^13^C DEPT Q NMR (125 MHz, pyridine-d_5_/D_2_O (250/10) with 0.2% TFA) spectrum of compound **1**., Figure S3: HSQC (pyridine-d_5_/D_2_O (250/10) with 0.2% TFA) spectrum of compound **1**, Figure S4: H2BC (pyridine-d_5_/D_2_O (250/10) with 0.2% TFA) spectrum of compound **1**., Figure S5: HMBC (pyridine-d_5_/D_2_O (250/10) with 0.2% TFA) spectrum of compound **1**., Figure S6: COSY (pyridine-d_5_/D_2_O (250/10) with 0.2% TFA) spectrum of compound **1**., Figure S7: T-ROESY (pyridine-d_5_/D_2_O (250/10) with 0.2% TFA) spectrum of compound **1**, Figure S8: TOCSY (pyridine-d_5_/D_2_O (250/10) with 0.2% TFA) spectrum of compound **1**. Figure S9: UPLC (TIC) chromatogram of compound **1**, Figure S10: ESI QTOF-MS and MS/MS spectra (negative ion mode) of compound **1**, Figure S11: ESI QTOF-MS and MS/MS spectra (positive ion mode) of compound **1**, Figure S12: HR-ESI-MS spectrum (positive ion mode) of compound **1**, Figure S13: HR-ESI-MS spectrum of compound **1**, Figure S14: Calculated formula for compound **1**, Figure S15: ^1^H NMR (500 MHz, methanol-d_4_) spectrum of compound **2**, Figure S16: ^13^C NMR (125 MHz, methanol-d4) spectrum of compound **2**, Figure S17: UPLC (TIC) chromatogram of compound **2**, Figure S18: ESI QTOF-MS (positive and negative ion mode) of compound **2**, Figure S19: ESI QTOF-MS/MS spectra (negative and positive ion mode) of compound **2**, Figure S20: HR-ESI-MS spectrum for compound **2**, Table S1. ^1^H (500 MHz) and ^13^C (125 MHz) NMR spectral data (δ ppm) for saponin 2 (CD_3_OD).
