# Supplementary material for: Serjanic Acid Glycosides from Chenopodium hybridum L. with Good Cytotoxicity and Selectivity Profile against Several Panels of Human Cancer Cell Lines
Source: Molecules. 2021 Aug 13;26(16):4915. doi: 10.3390/molecules26164915 (PMC8401257; doi:10.3390/molecules26164915)
Supplement: Supplementary file 1 [file molecules-26-04915-s001.zip › molecules-1330089-supplementary.pdf]

Supplementary Materials:

Title: Serjanic acid glycosides from Chenopodium hybridum L. with good cytotoxicity and selectivity profile against several panels of human cancer cell lines.

Karolina Grabowska[1], Łukasz Pecio[2], Agnieszka Galanty[1], Paweł Żmudzki[3], Wiesław Oleszek[2] and Irma Podolak[1]*

[1]Department of Pharmacognosy, Jagiellonian University Medical College, 9 Medyczna str., 30-688 Cracow, Poland

[2]Department of Biochemistry and Crop Quality, Institute of Soil Science and Plant Cultivation – State Research Institute, ul. Czartoryskich 8, 24-100, Puławy, Poland

[3]Department of Pharmaceutical Chemistry, Jagiellonian University Medical College, 9 Medyczna str., 30-688 Cracow, Poland

* Correspondence: irma.podolak@uj.edu.pl

Table of contents:

Figure S1.^{1}H NMR (500 MHz, pyridine-d_5/D_2O (250/10) with 0.2% TFA) spectrum of compound 1
Figure S2. ^{13}C DEPT Q NMR (125 MHz, pyridine-d_5/D_2O (250/10) with 0.2% TFA) spectrum of compound 1.
Figure S3. HSQC (pyridine-d_5/D_2O (250/10) with 0.2% TFA) spectrum of compound 1
Figure S4. H2BC (pyridine-d_5/D_2O (250/10) with 0.2% TFA) spectrum of compound 1.
Figure S5. HMBC (pyridine-d_5/D_2O (250/10) with 0.2% TFA) spectrum of compound 1.
Figure S6. COSY (pyridine-d_5/D_2O (250/10) with 0.2% TFA) spectrum of compound 1.
Figure S7. T-ROESY (pyridine-d_5/D_2O (250/10) with 0.2% TFA) spectrum of compound 1
Figure S8. TOCSY (pyridine-d_5/D_2O (250/10) with 0.2% TFA) spectrum of compound 1.
Figure S9. UPLC (TIC) chromatogram of compound 1.
Figure S10. ESI QTOF-MS and MS/MS spectra (negative ion mode) of compound 1.
Figure S11. ESI QTOF-MS and MS/MS spectra (positive ion mode) of compound 1.
Figure S12. HR-ESI-MS spectrum (positive ion mode) of compound 1.
Figure S13. HR-ESI-MS spectrum of compound 1.
Figure S14. Calculated formula for compound 1.
Figure S15. ^{1}H NMR (500 MHz, methanol-d_4) spectrum of compound 2
Figure S16. ^{13}C NMR (125 MHz, methanol-d_4) spectrum of compound 2
Figure S17. UPLC (TIC) chromatogram of compound 2.
Figure S18. ESI QTOF-MS (positive and negative ion mode) of compound 2.
Figure S19. ESI QTOF-MS/MS spectra (negative and positive ion mode) of compound 2.
Figure S20. HR-ESI-MS spectrum for compound 2.
Table S1. ^{1}H (500 MHz) and ^{13}C (125 MHz) NMR spectral data (δ ppm) for saponin **2** (CD_3OD).

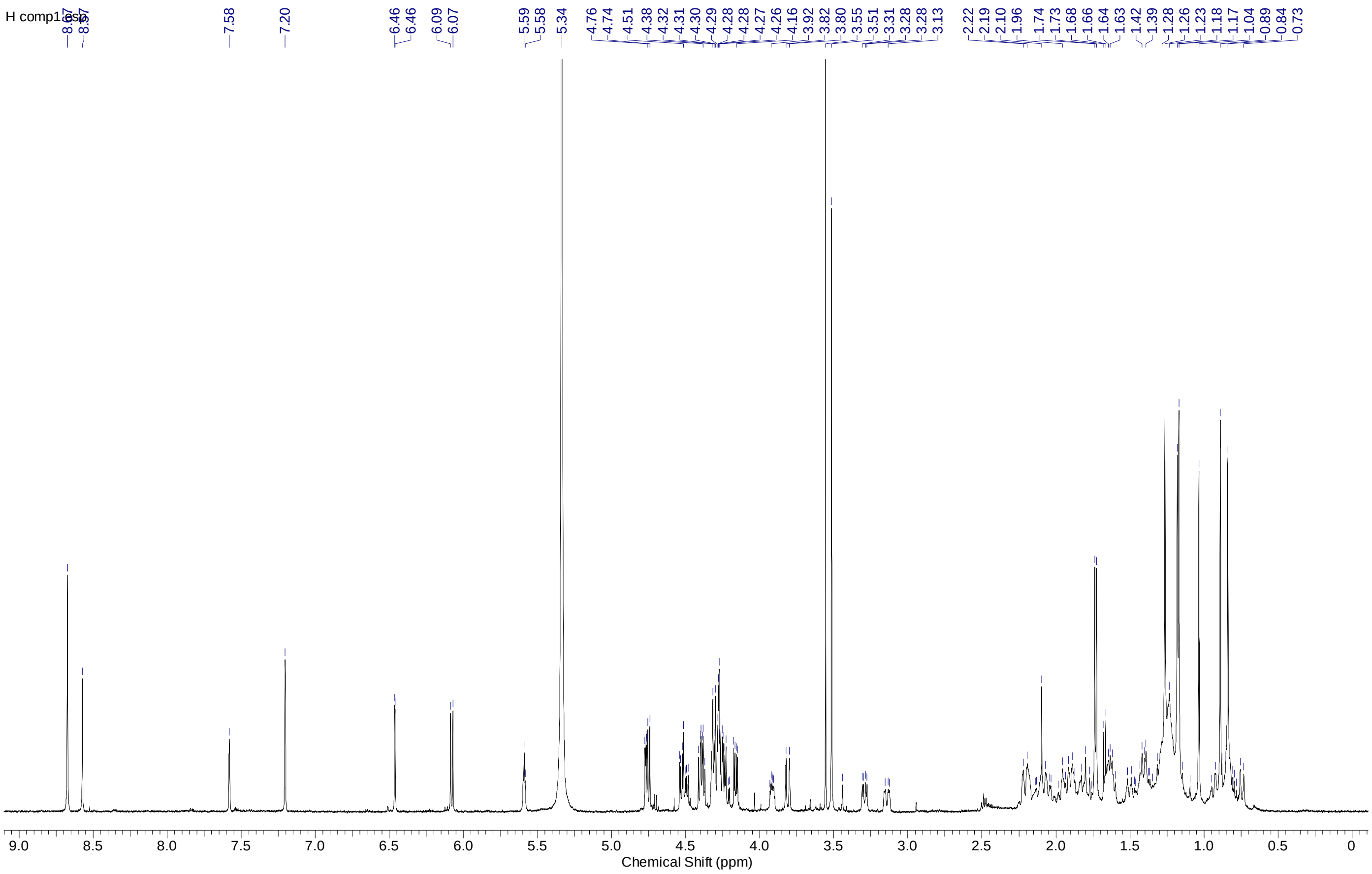

Figure S1. ^{1}H NMR (500 MHz, pyridine-d_5/D_2O (250/10) with 0.2% TFA) spectrum of compound 1.

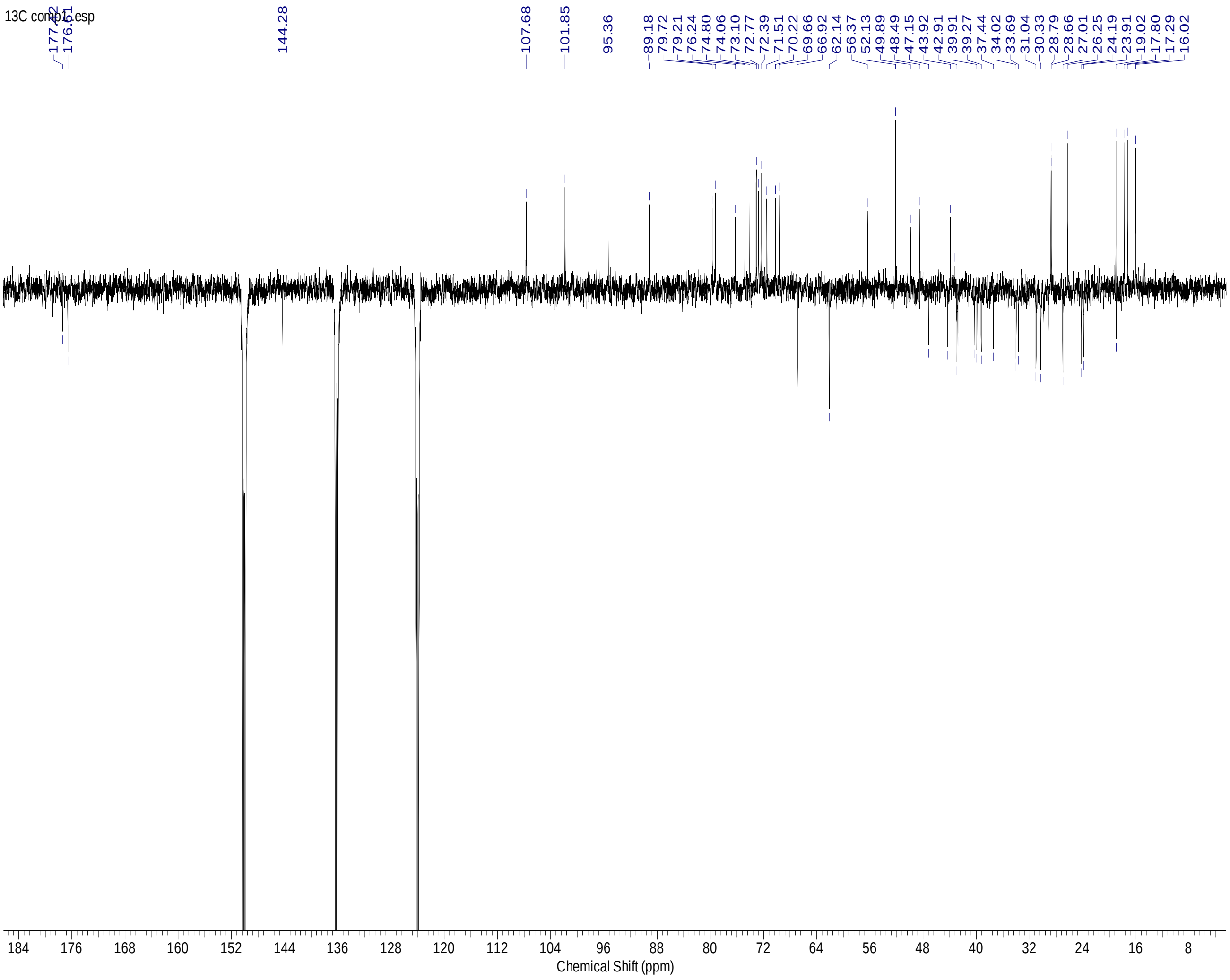

Figure S2. ^{13}C DEPT Q NMR (125 MHz, pyridine-d$_5$/D_2O (250/10) with 0.2% TFA) spectrum of compound 1.

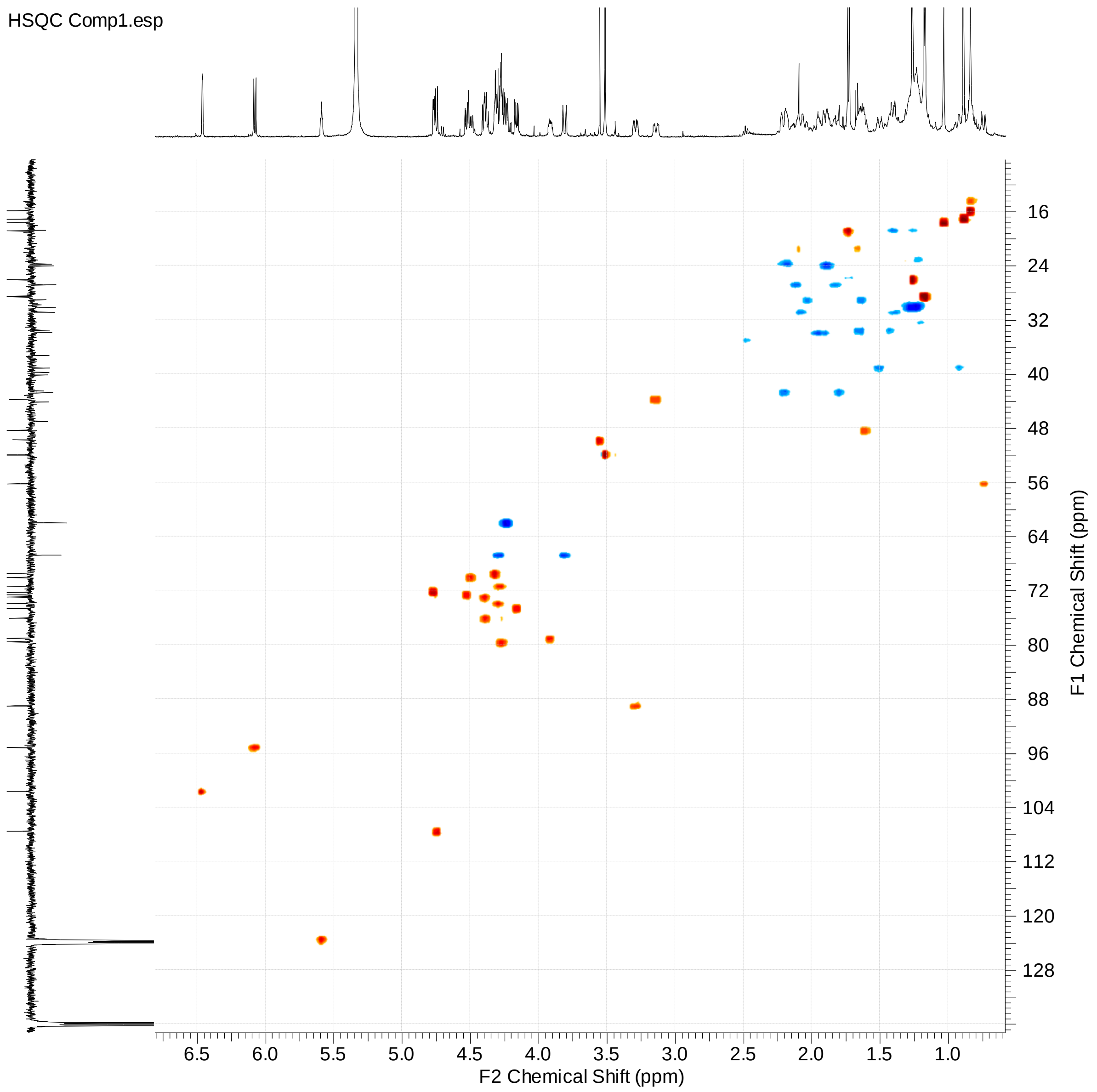

Figure S3. HSQC (pyridine-d_5/D_2O (250/10) with 0.2% TFA) spectrum of compound 1.

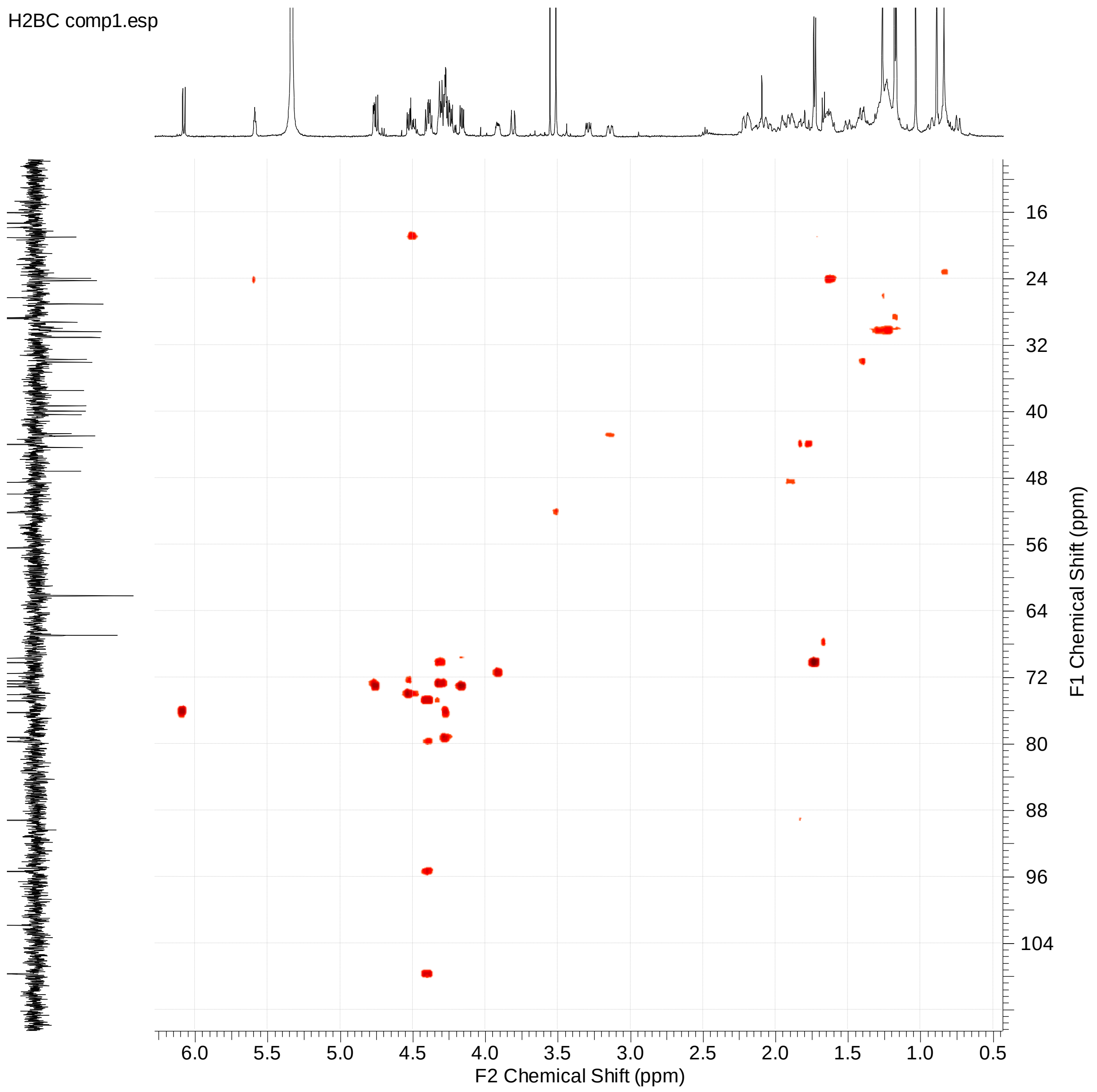

Figure S4. H2BC (pyridine-d_5/D_2O (250/10) with 0.2% TFA) spectrum of compound 1.

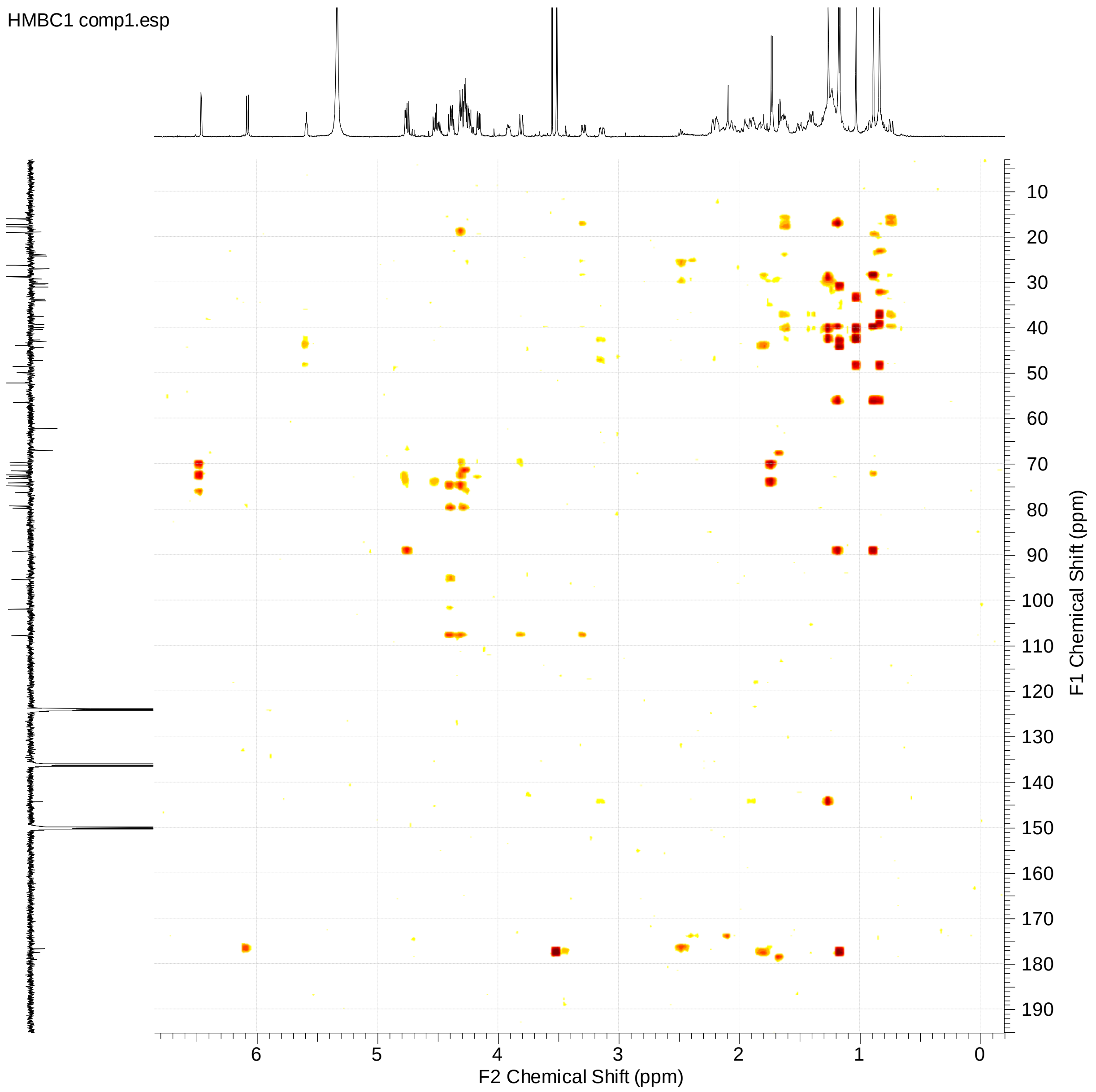

Figure S5. HMBC (pyridine-d_5/D_2O (250/10) with 0.2% TFA) spectrum of compound 1.

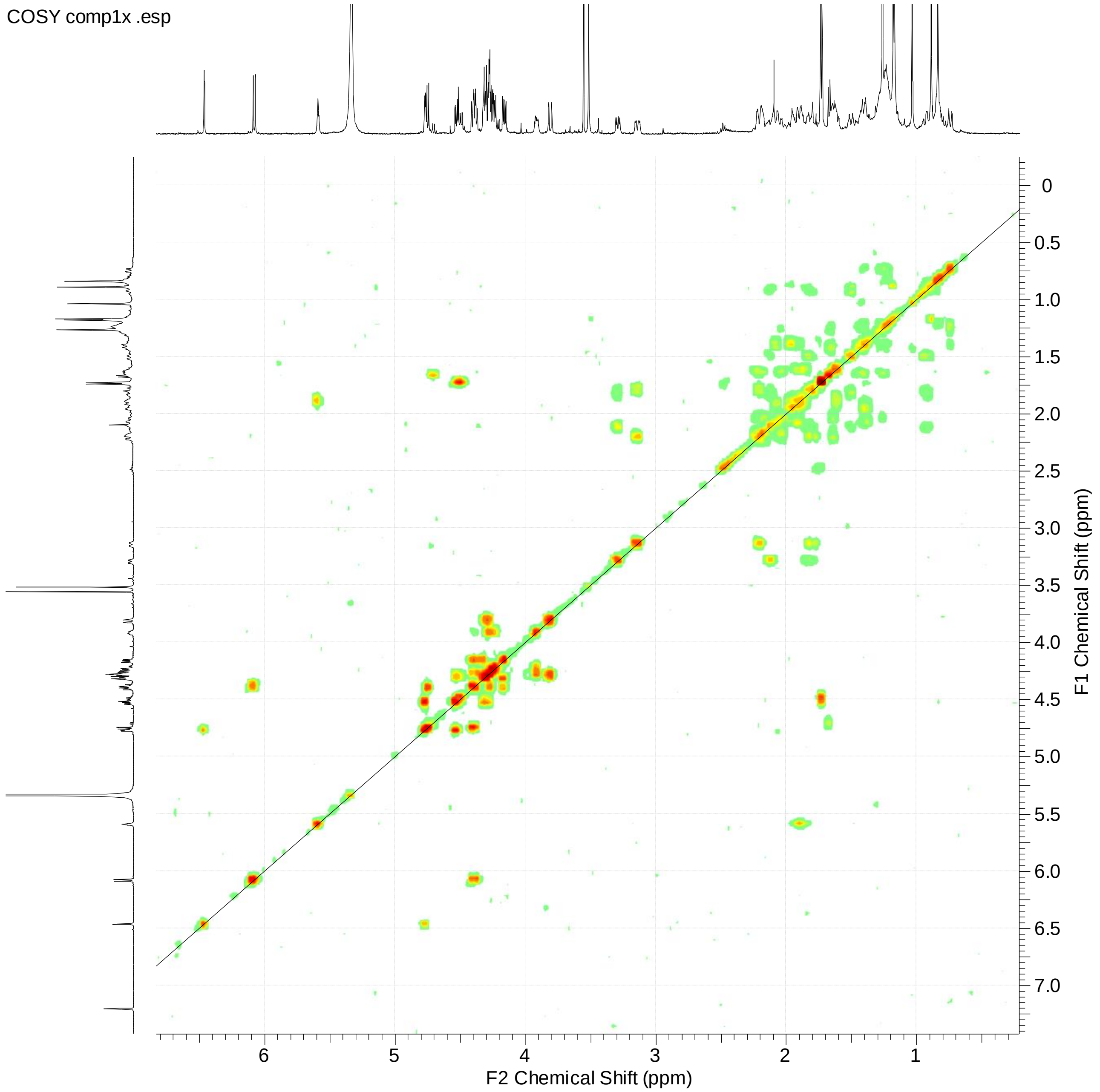

Figure S6. COSY (pyridine-d_5/D_2O (250/10) with 0.2% TFA) spectrum of compound 1.

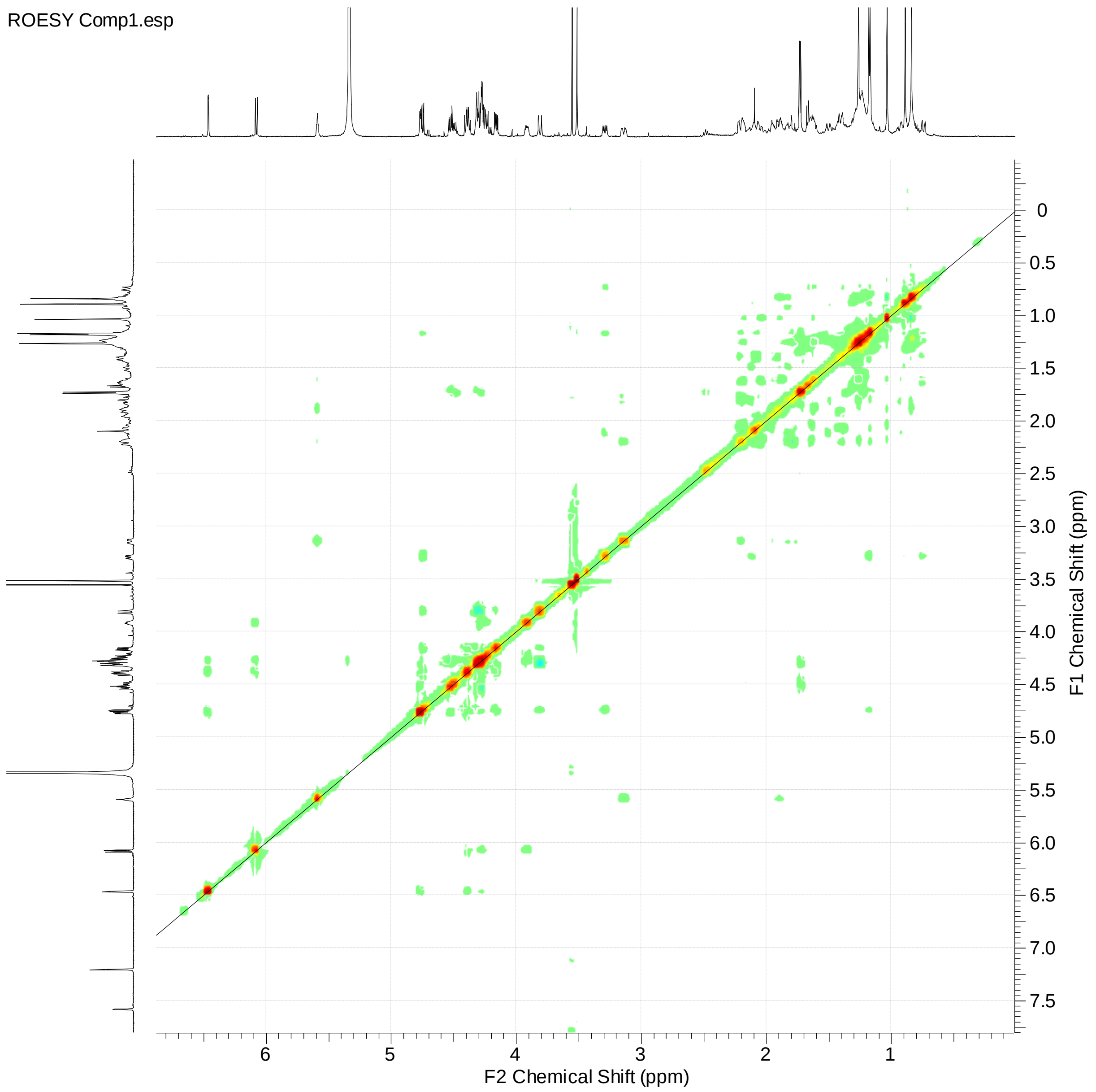

Figure S7. T-ROESY (pyridine-d_5/D_2O (250/10) with 0.2% TFA) spectrum of compound 1.

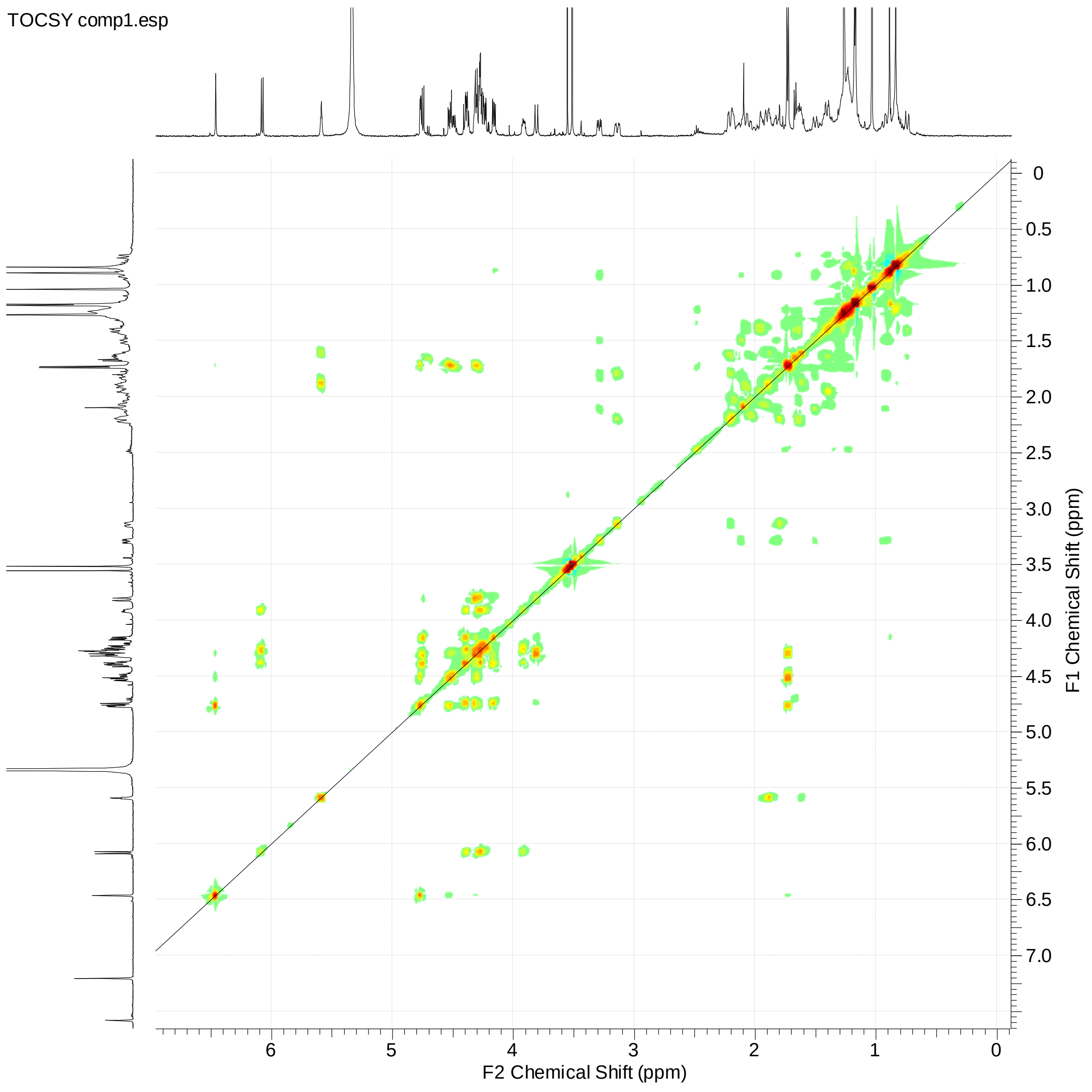

Figure S8. TOCSY (pyridine-d_5/D_2O (250/10) with 0.2% TFA) spectrum of compound 1.

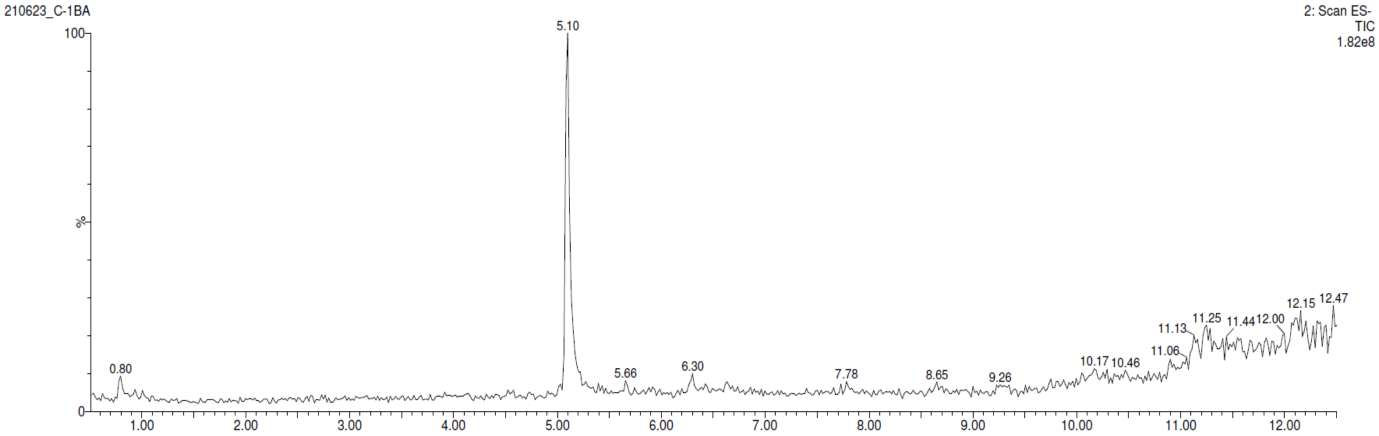

Figure S9. UPLC (TIC) chromatogram of compound 1.

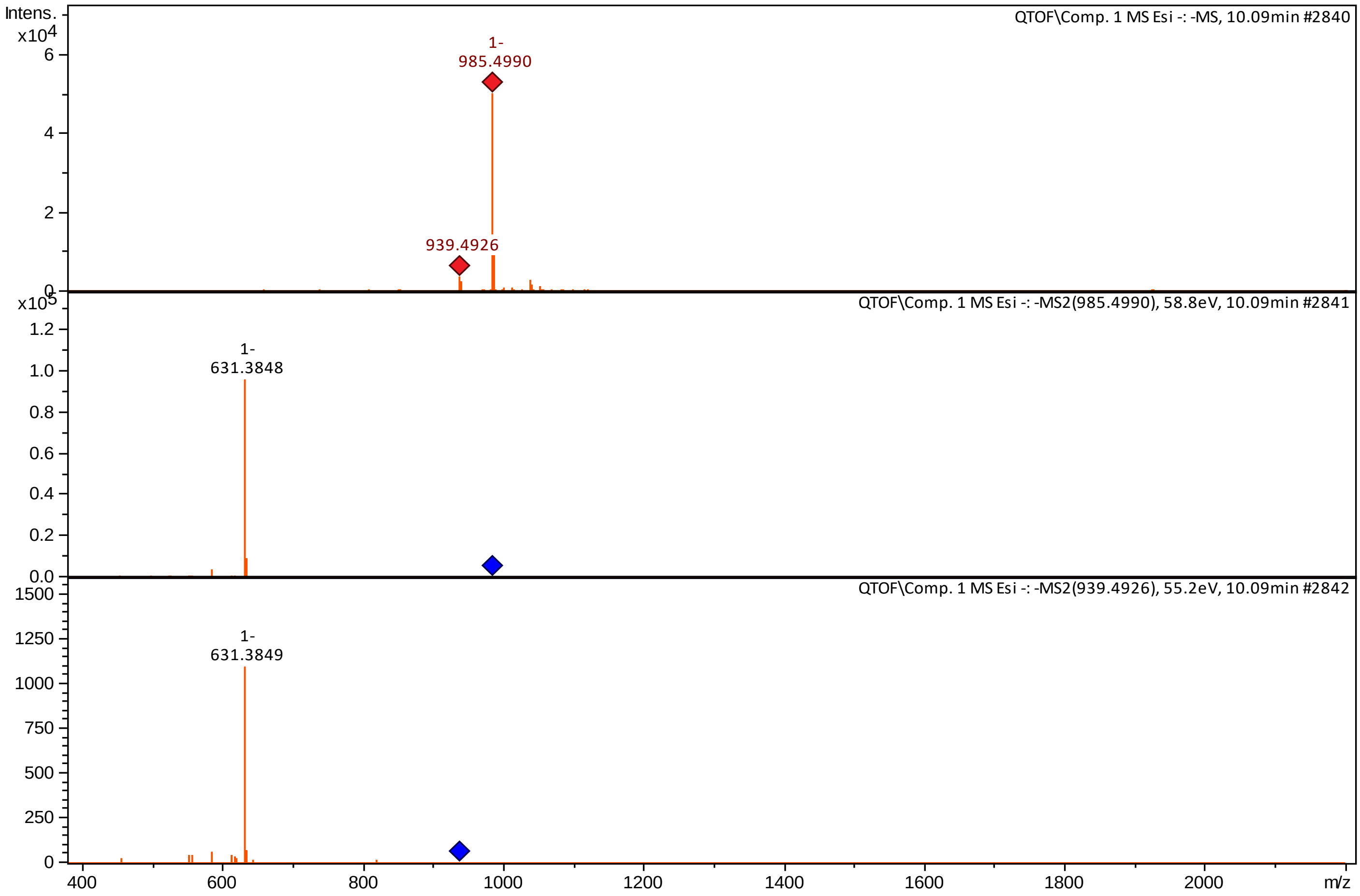

Figure S10. ESI QTOF-MS and MS/MS spectra (negative ion mode) of compound 1.

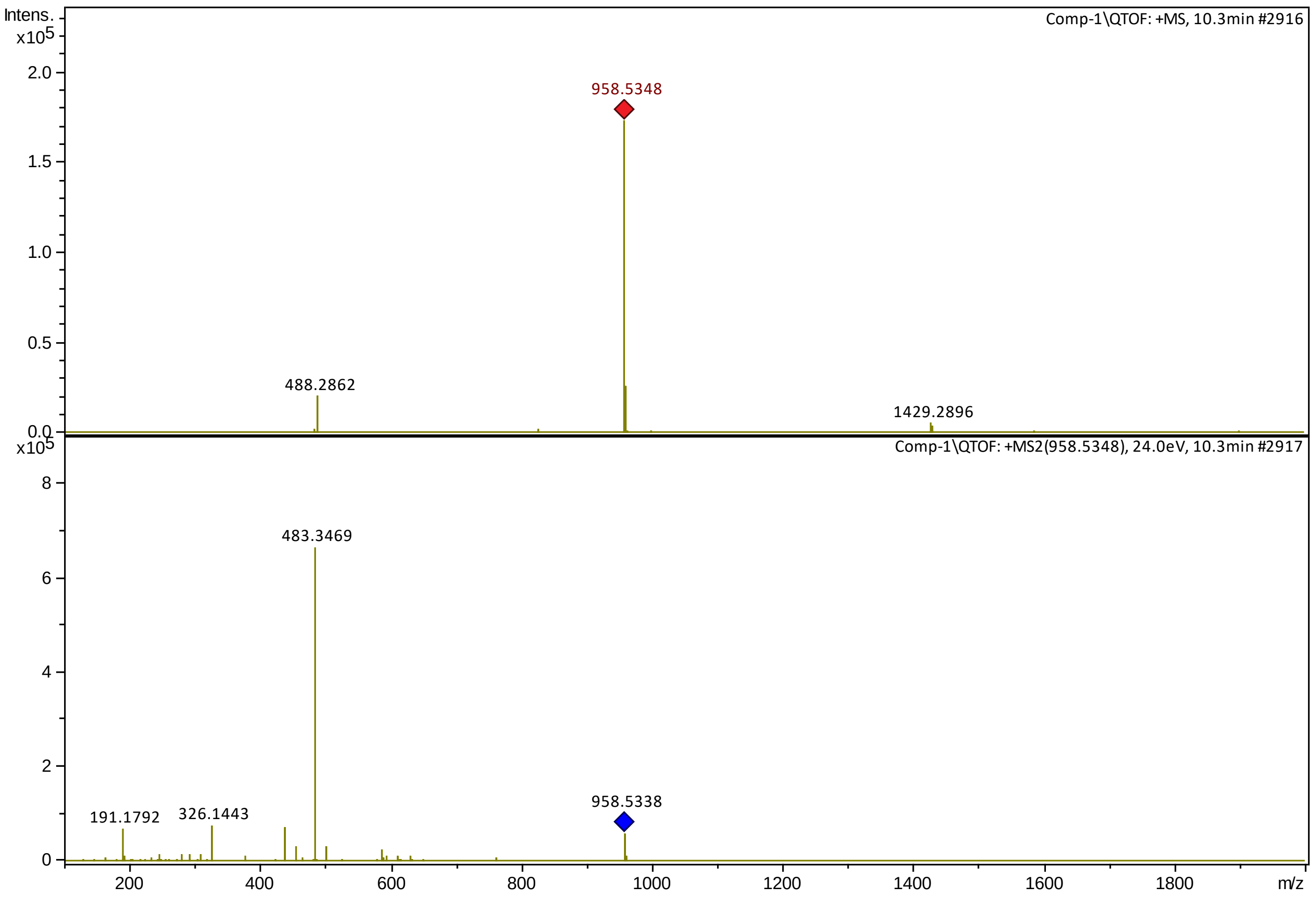

Figure S11. ESI QTOF-MS and MS/MS spectra (positive ion mode) of compound 1.

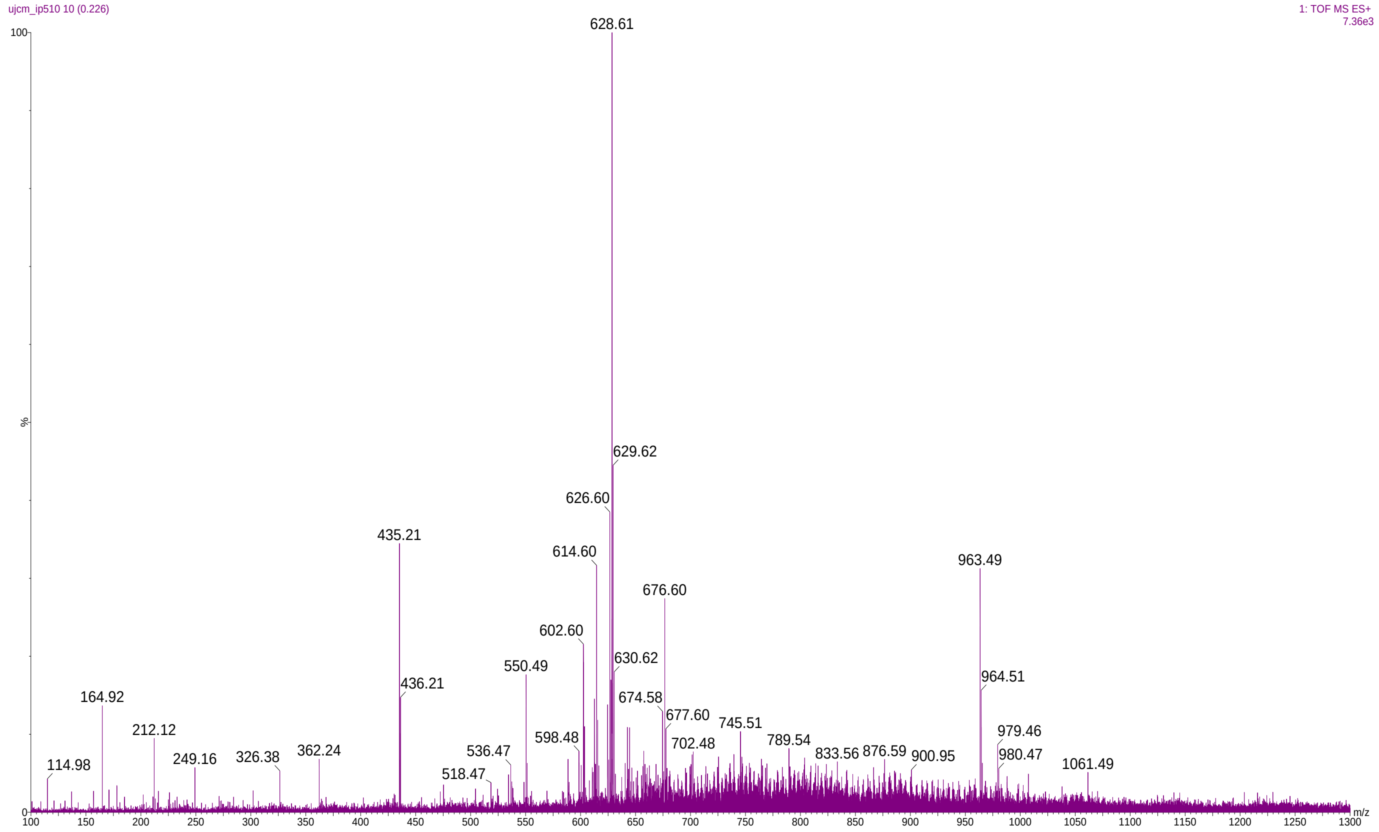

Figure S12. HR-ESI-MS spectrum (positive ion mode) of compound 1.

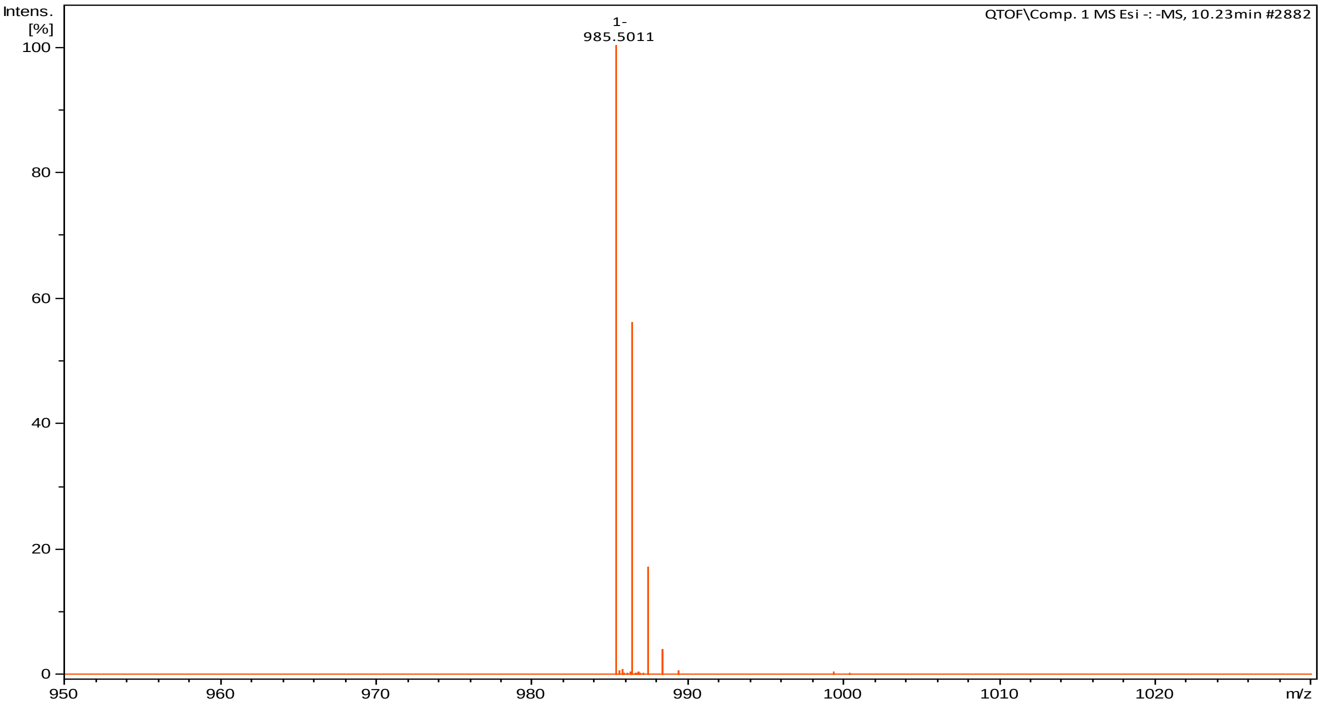

Figure S13. HR-ESI-MS- spectrum of compound 1.

SmartFormula Manually

Lower: C_{42}

Upper: N_0

C 42-n, N 0-0

Note: for m < 2000 the elements C, H, N, and O are considered implicitly.

Adducts, pos.:

☑ Collect adducts

Adducts, neg.: M-H; M+HCOOH-H

Measured: 985.5011 Tolerance: 2 mDa Charge: -1

Meas. m/z	Adduct	#	Ion Formula	Score	m/z	err [mDa]	err [ppm]	mSigma	rdb	e— Conf	N-Rule	H/C	O/C	Sum Formula
985.5011	M-H	1	$C_{49}H_{77}O_{20}$	94.88	985.5014	0.3	0.3	9.5	11.0	even	ok	1.571	0.408	$C_{49}H_{78}O_{20}$
985.5011	M+HCOOH-H	1	$C_{49}H_{77}O_{20}$	100.00	985.5014	0.3	0.3	9.5	11.0	even	ok	1.571	0.408	$C_{48}H_{76}O_{18}$
939.4953	M-H	1	$C_{48}H_{75}O_{18}$	100.00	939.4959	0.6	0.6	7.6	11.0	even	ok	1.563	0.375	$C_{48}H_{76}O_{18}$
1031.5068	M+HCOOH-H	1	$C_{50}H_{79}O_{22}$	94.88	1031.5068	0.0	0.0	n.a.	11.0	even	ok	1.580	0.440	$C_{49}H_{78}O_{20}$

Figure S14. Calculated formula for compound 1.

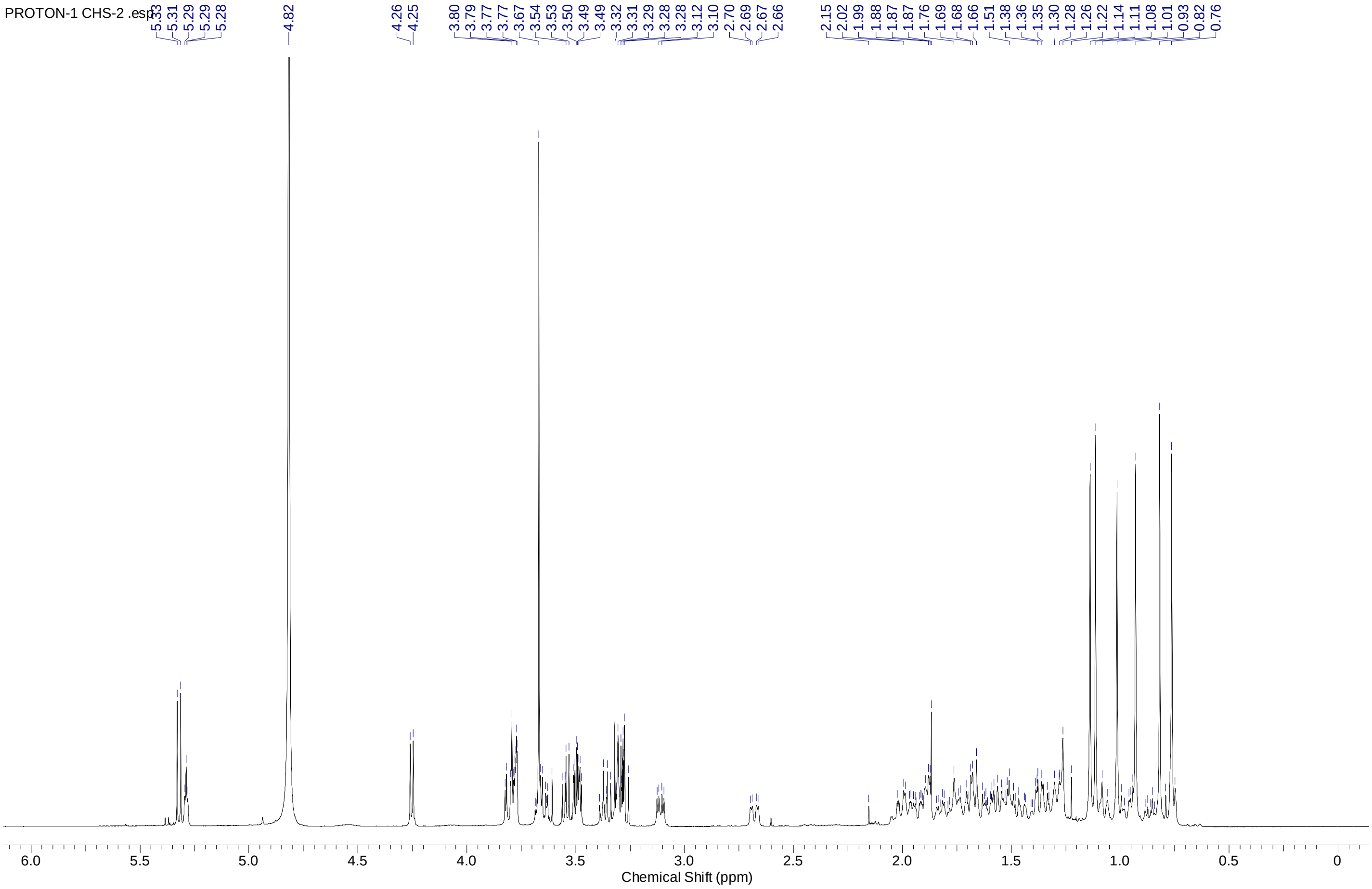

Figure S15. The ^{1}H NMR (500 MHz, CD_3OD) spectrum of compound 2.

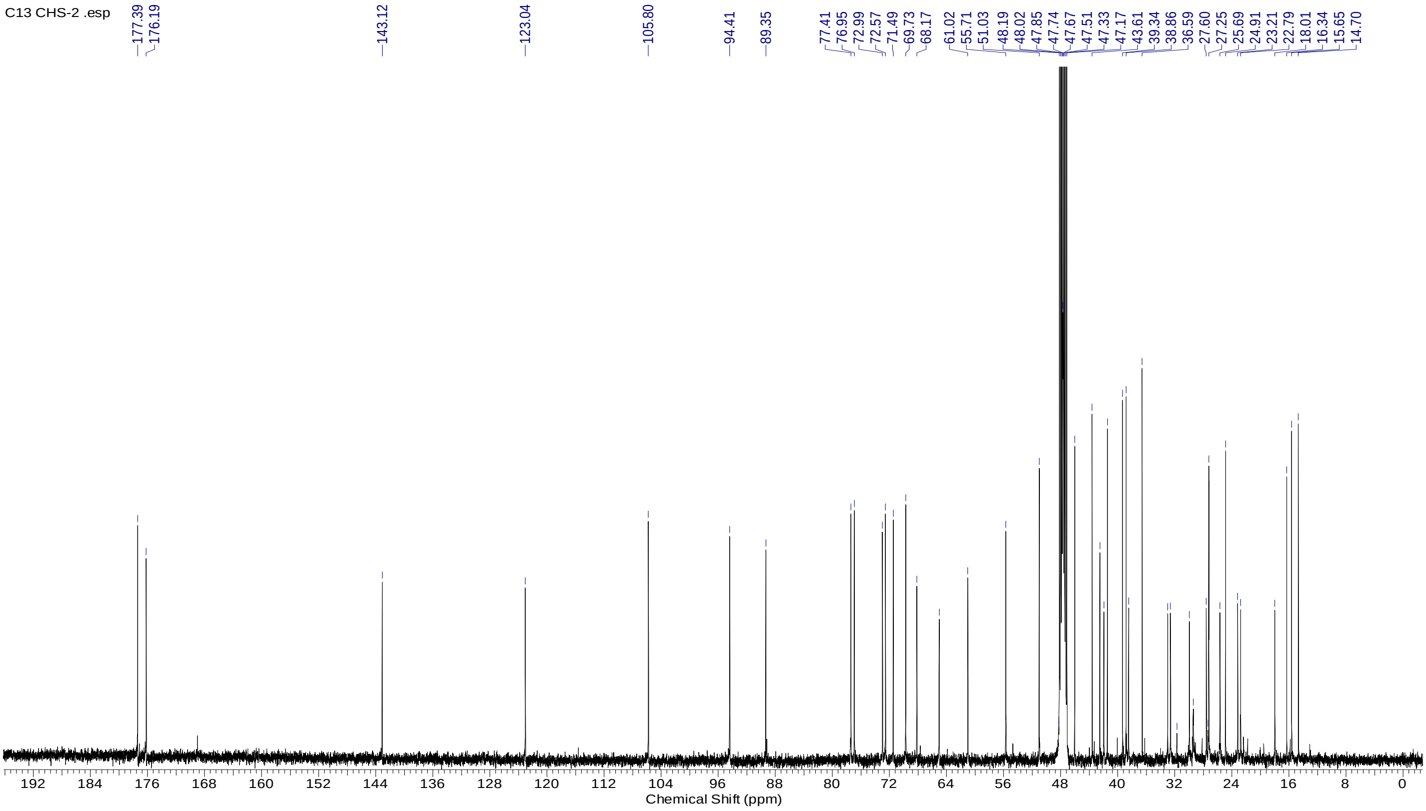

Figure S16. The ^{13}C NMR (125 MHz, CD_3OD) spectrum of compound 2.

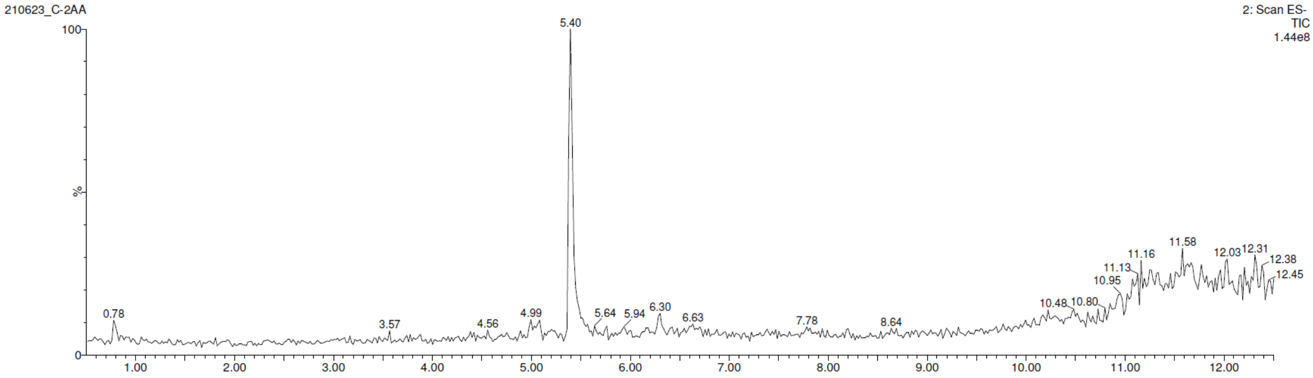

Figure S17. UPLC (TIC) chromatogram of compound 2.

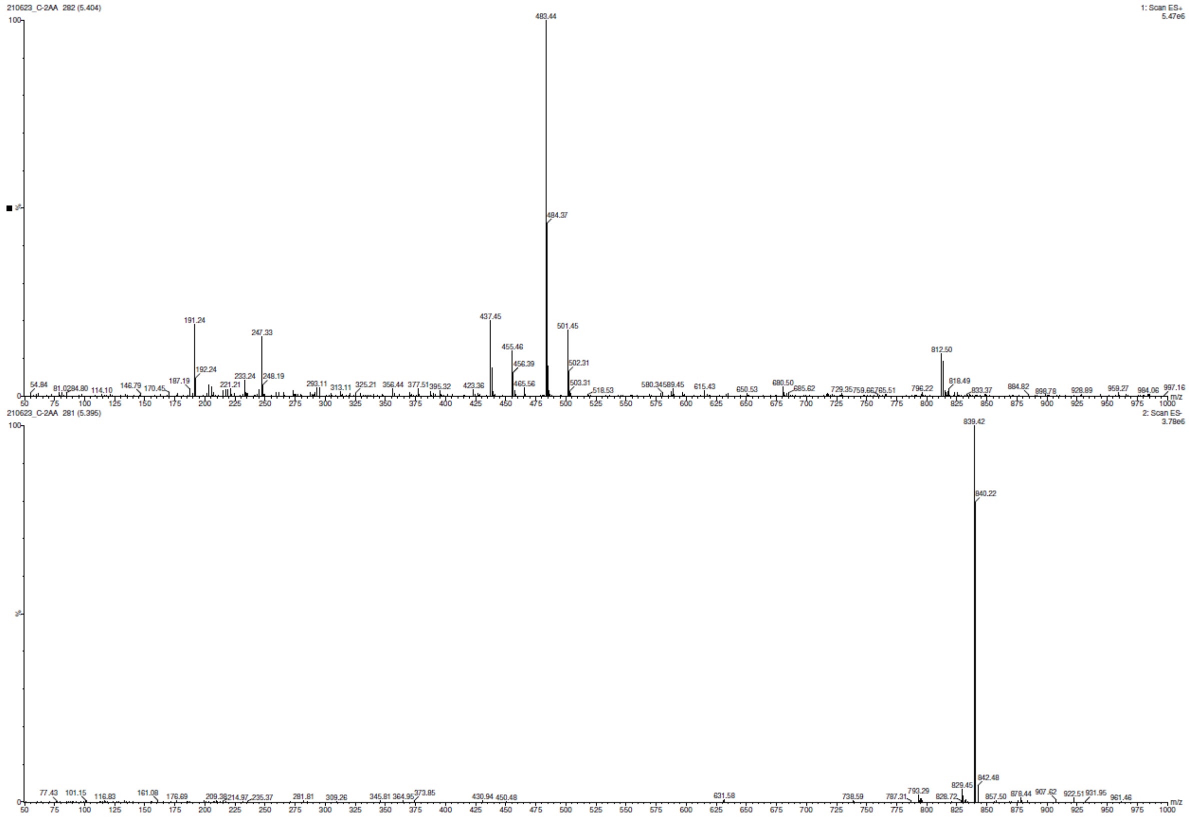

Figure S18. ESI QTOF-MS (positive and negative ion mode) of compound 2.

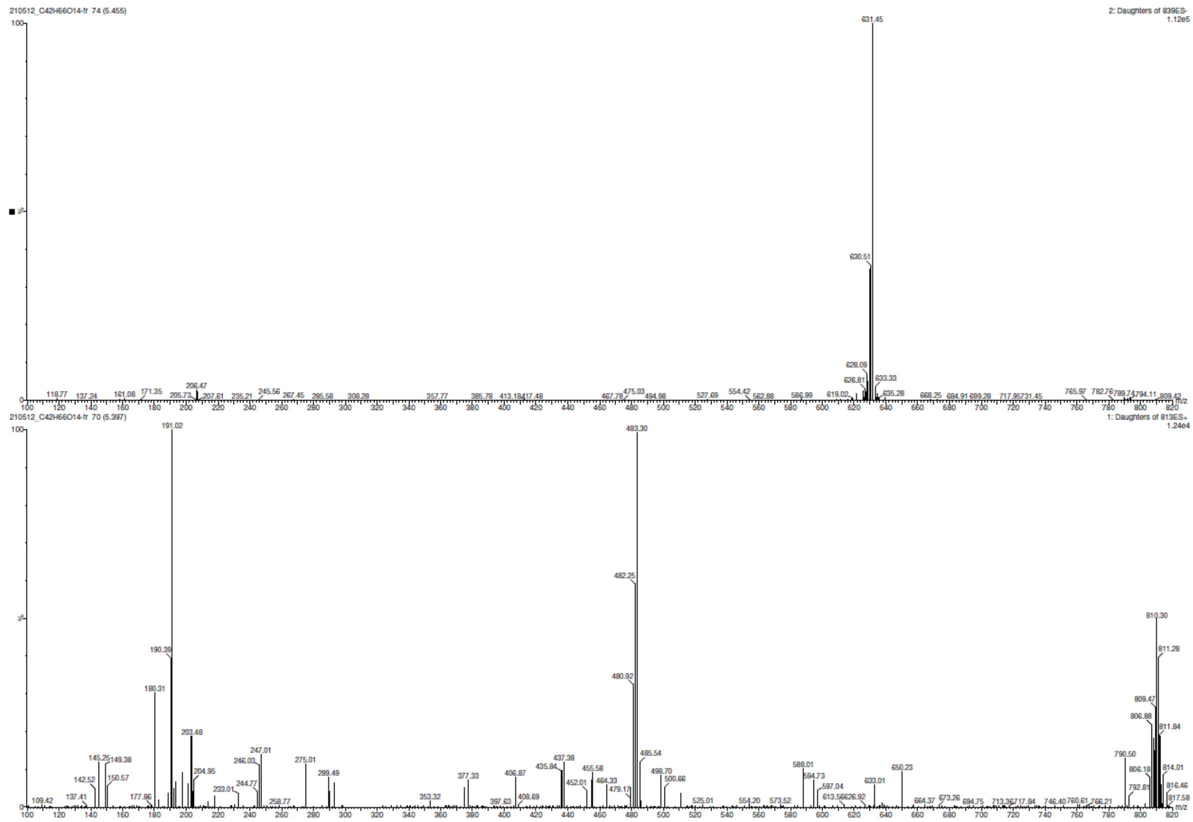

Figure S19. ESI QTOF-MS/MS spectra (negative and positive ion mode) of compound 2.

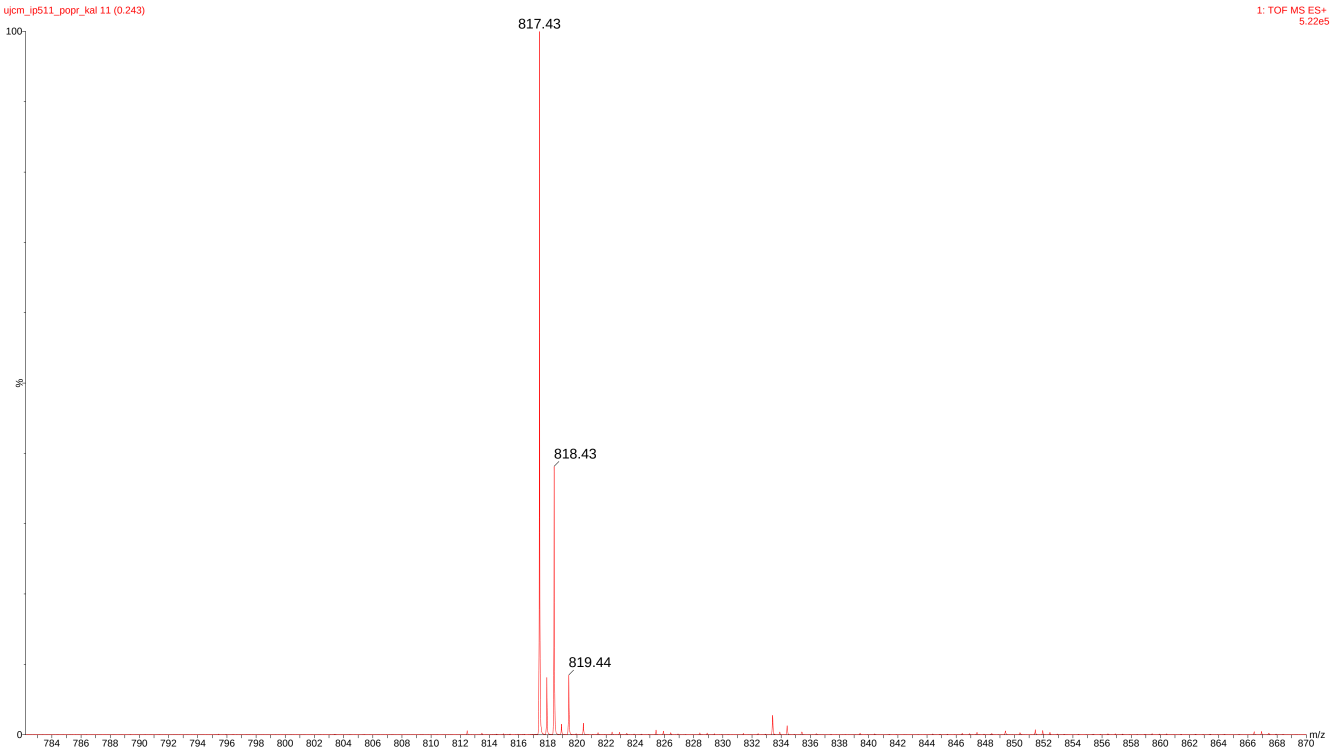

Figure S20. HR-ESI-MS spectrum for compound 2.

Table S1. ^{1}H (500 MHz) and ^{13}C (MHz) NMR spectral data (δ ppm) for saponin **2** (CD_3OD)

No.	δC	δH (*J* in Hz)*
1	38.5	0.95, 1.60
2	25.7	1.67, 1.82
3	89.3	3.11 dd (11.7, 4.5)
4	38.9	-
5	55.7	0.76
6	18.0	1.38, 1.53
7	32.7	1.30, 1.46
8	39.3	-
9	47.7	1.57
10	36.6	-
11	23.2	1.87
12	123.0	5.29 t (3.7)
13	143.1	-
14	41.5	-
15	27.6	1.06, 1.76
16	22.7	1.75, 2.0
17	46.0	-
18	42.5	2.7
19	41.9	1.65, 1.93
20	43.6	-
21	29.9	1.35, 1.98
22	33.0	1.50, 1.69
23	27.3	1.01 s
24	15.6	0.82 s
25	14.7	0.93 s
26	16.3	0.77 s
27	24.9	1.14 s
28	176.1	-
29	27.2	1.11 s
30	177.4	-
-OCH3	51.0	3.67 s
3-*O*-α-L-Ara		
1	105.8 1	4.25 d (6.8)
2	71.4	3.53
3	73.0	3.48
4	68.1	3.78
5	65.0	3.49<br>3.81
28-*O*-β-D-Glc		
1	94.4 1	5.32 d (8.0)
2	72.5	3.28
3	76.9	3.37
4	69.7	3.31
5	77.4	3.31
6	61.0	3.64<br>3.78

*Overlapping signals are reported without designated multiplicity
